# Desynchronization of Neocortical Networks by Asynchronous Release of GABA at Autaptic and Synaptic Contacts from Fast-Spiking Interneurons

**DOI:** 10.1371/journal.pbio.1000492

**Published:** 2010-09-28

**Authors:** Frédéric Manseau, Silvia Marinelli, Pablo Méndez, Beat Schwaller, David A. Prince, John R. Huguenard, Alberto Bacci

**Affiliations:** 1 European Brain Research Institute, Rome, Italy; 2 Unit of Anatomy, Department of Medicine, University of Fribourg, Fribourg, Switzerland; 3 Department of Neurology and Neurological Sciences, Stanford University School of Medicine, Stanford, California, United States of America; Northwestern University, United States of America

## Abstract

Networks of specific inhibitory interneurons regulate principal cell firing in several forms of neocortical activity. Fast-spiking (FS) interneurons are potently self-inhibited by GABAergic autaptic transmission, allowing them to precisely control their own firing dynamics and timing. Here we show that in FS interneurons, high-frequency trains of action potentials can generate a delayed and prolonged GABAergic self-inhibition due to sustained asynchronous release at FS-cell autapses. Asynchronous release of GABA is simultaneously recorded in connected pyramidal (P) neurons. Asynchronous and synchronous autaptic release show differential presynaptic Ca^2+^ sensitivity, suggesting that they rely on different Ca^2+^ sensors and/or involve distinct pools of vesicles. In addition, asynchronous release is modulated by the endogenous Ca^2+^ buffer parvalbumin. Functionally, asynchronous release decreases FS-cell spike reliability and reduces the ability of P neurons to integrate incoming stimuli into precise firing. Since each FS cell contacts many P neurons, asynchronous release from a single interneuron may desynchronize a large portion of the local network and disrupt cortical information processing.

## Abstract 2

An activity-dependent long-lasting asynchronous release of GABA from identified fast-spiking inhibitory neurons in the neocortex can impair the reliability and temporal precision of activity in a cortical network.

## Author Summary

In the cerebral cortex (neocortex) of the brain, fast-spiking (FS) inhibitory cells contact many principal pyramidal (P) neurons on their cell bodies, which allows the FS cells to control the generation of action potentials (neuronal output). FS-cell-mediated rhythmic and synchronous inhibition drives coherent network oscillations of large ensembles of P neurons, indicating that FS interneurons are needed for the precise timing of cortical circuits. Interestingly, FS cells are self-innervated by GABAergic autaptic contacts, whose synchronous activation regulates FS-cell precise firing. Here we report that high-frequency firing in FS interneurons results in a massive (>10-fold), delayed, and prolonged (for seconds) increase in inhibitory events, occurring at both autaptic (FS–FS) and synaptic (FS–P) sites. This increased inhibition is due to asynchronous release of GABA from presynaptic FS cells. Delayed and disorganized asynchronous inhibitory responses significantly affected the input–output properties of both FS and P neurons, suggesting that asynchronous release of GABA might promote network desynchronization. FS interneurons can fire at high frequency (>100 Hz) in vitro and in vivo, and are known for their reliable and precise signaling. Our results show an unprecedented action of these cells, by which their tight temporal control of cortical circuits can be broken when they are driven to fire above certain frequencies.

## Introduction

In the cerebral cortex, the control of neuronal population discharge pattern and timing is of fundamental importance for information processing and cognitive operations [1],[2]. Remarkably, cortical neurons have a variety of means to precisely control their spike timing, either through their own intrinsic membrane properties [3],[4] or through highly coordinated interactions with recurrent networks of local GABA-releasing (GABAergic) inhibitory neurons [5]–[8].

Distinct cortical interneuron classes have a wide range of preferred firing patterns [9],[10] that result in diverse tuning properties, important for setting network dynamics [11]. In addition to their firing properties, interneuron-specific patterns of axonal projections are also critical in determining GABA-mediated effects on pyramidal (P) cells. Indeed, cortical interneurons can be divided into two major functional types: those that innervate the dendrites of P cells, mainly controlling their information processing and integration, and those that target the P-neuron perisomatic region, thus controlling the output and, most notably, the precision of spike timing in large principal-cell populations [12]–[14].

Despite the large heterogeneity of cortical inhibitory neurons, the main population of perisomatic-targeting fast-spiking (FS) interneurons is relatively homogeneous throughout the cerebral cortex. Several factors likely contribute to making these interneurons highly specialized for the control of P spike precision, including their short membrane time constants; intrinsic excitability [15]; the presence of Kv3 potassium channels, which efficiently accelerate the repolarization of action potentials (APs) [16]; sub-millisecond AMPA receptor conductances [15],[17]–[19]; the rapid and reliable synchronous release of GABA at their terminals [20]–[23]; the almost ubiquitous expression of the Ca^2+^-binding protein parvalbumin (PV) in these cells; and the characteristic firing of APs at high constant rate with no adaptation. Another factor that distinguishes FS cells from other interneuron types is their hypothesized rigid (non-plastic) action as precision devices during cortical operations, leading to the view that they represent a dedicated system for regulating the timing of activity in neocortical circuits [24],[25]. A fundamental question remaining, however, is whether this synchronizing function of FS interneurons could turn into a desynchronizing action under certain conditions, such as during asynchronous release of GABA.

Presynaptic terminals at several synapses respond to trains of APs with a delayed and long-lasting asynchronous release of synaptic vesicles [20],[26]–[28]. This form of activity-dependent increase in spontaneous transmitter release has been studied intensely in experiments designed to characterize the molecular events underlying synaptic vesicle release processes and plasticity [29]. It occurs especially during early neuronal development [30], and its magnitude is generally small compared to synchronous, single-spike-dependent release [31],[32]. Interestingly, a prominent asynchronous release of GABA from basket cells onto P neurons is present in the hippocampus, where it appears to be selective for CCK-positive but not PV-containing basket cells [20],[33].

FS cells are self-innervated to a great extent by GABAergic autapses [22],[34] that control the spike-timing precision and firing dynamics of these neurons [35]. Interestingly, FS basket cells can fire at very high frequencies (>200 Hz) in cortical slices and in vivo in response to intracellular depolarization [36]–[41] or network activity [41]–[43]. In rodents, in vivo, neocortical FS interneurons fire bursts of APs closely matching the periodicity of “very fast oscillations” (400–600 Hz) triggered by vibrissae stimulation [44],. Thus, this very high frequency firing could, in principle, induce asynchronous synaptic release.

Here we show that trains of APs in FS cells elicit asynchronous GABA release that can persist for several seconds at both autaptic and FS–P synapses and can be blocked by increasing slow intracellular calcium buffering. Although asynchronous release was proposed to change postsynaptic gain for a relatively prolonged period after presynaptic activity has subsided [20],[28],[31],[33], a direct proof for this functional role of asynchronous release has remained elusive [27]. The present results indicate that asynchronous release of GABA alters firing efficacy of FS cells, as well as spike reliability and precision in postsynaptic P neurons. Since each FS interneuron contacts a number of neurons in the local circuit, asynchronous release of GABA from one FS cell might desynchronize a relatively large portion of the cortical network.

## Results

### Delayed and Self-Induced Increase of GABAergic Inhibition in FS Interneurons

**Figure 1:**
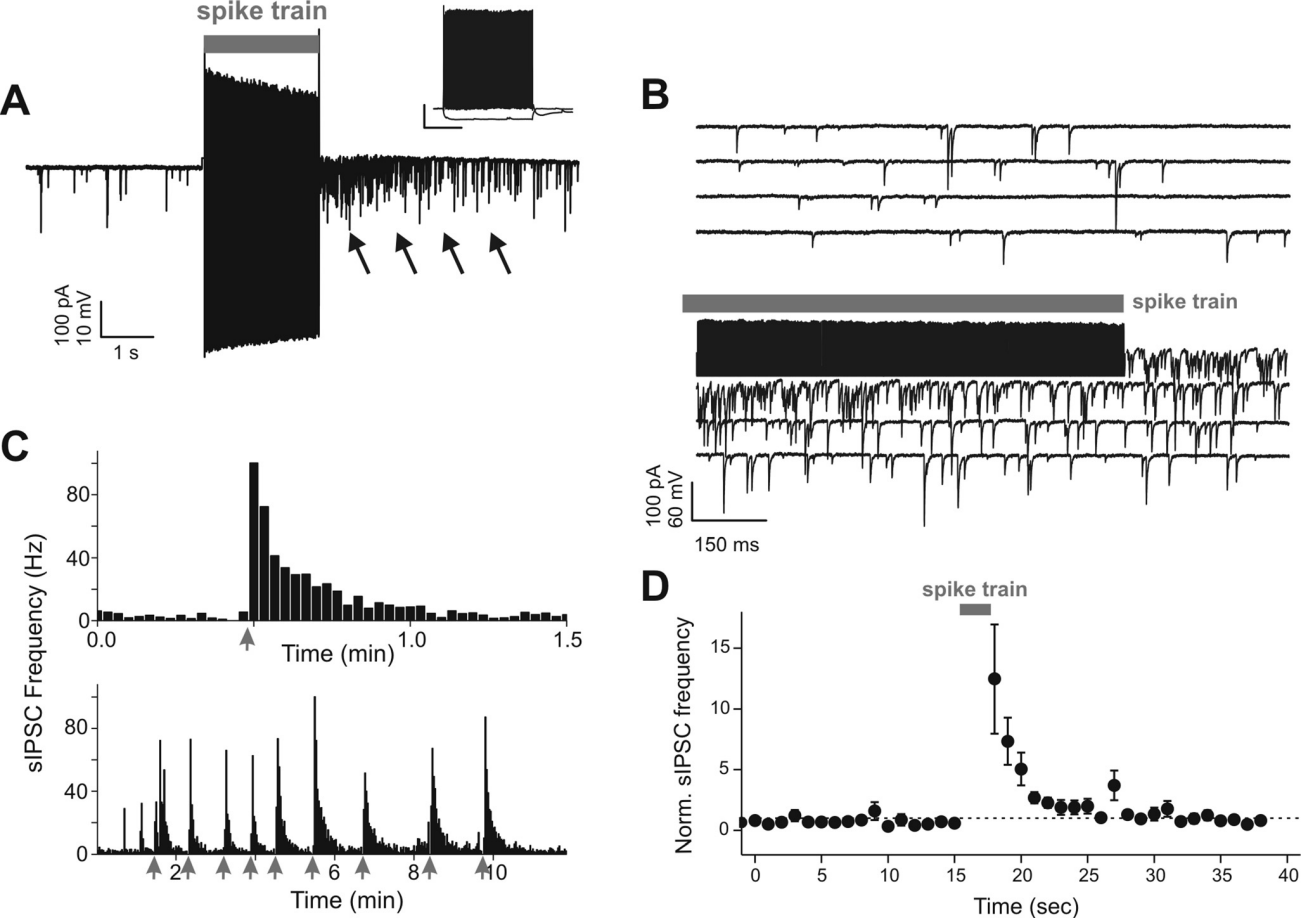
Delayed and self-induced increase of GABAergic inhibition in FS interneurons. (A) Continuous voltage-clamp recording from a FS interneuron showing IPSCs (sIPSCs) before and after an AP train (2 s) elicited in current-clamp (gray bar). Note that the train is followed by a sustained increase in sIPSC frequency, which lasts for several seconds (arrows). Inset: firing pattern of the cell of (A) in response to 600-ms current injections (−50 and 250 pA) revealing its FS behavior in response to a depolarization. Scale bars: 20 mV; 250 ms. (B) Same trace as in (A) shown at an expanded time scale. (C) Time course of sIPSC frequency of cell shown in (A), calculated in 10-s bins. Gray arrows indicate onset of spike trains. Note the persistent sIPSC frequency increase following a spike train (top panel) and the consistency of the response when elicited multiple times (bottom panel). (D) Average plot of normalized sIPSC frequency versus time (*n* = 11 cells).

We recorded from layer V FS interneurons in acute slices obtained from rat somatosensory cortex. FS interneurons were visually identified under infrared videomicroscopy as multipolar neurons lacking clear apical dendrites. During electrophysiological recordings, these cells typically showed FS firing behavior, namely brief APs and abrupt high-frequency firing (>40 Hz) in response to depolarizing direct current steps (Figure 1, inset) [22],.

When trains of APs were evoked in FS cells by depolarizing pulses, we often observed a large increase in the frequency of spontaneous inhibitory postsynaptic currents (sIPSCs) in the same neurons. This increased inhibition lasted for several seconds (Figure 1A, arrows). In our recording conditions (see Materials and Methods), sIPSCs occurred relatively rarely in FS neurons at rest (mean frequency = 11.7±3.3 Hz). However, a massive increase (>10-fold) in the incidence of these events was evident immediately after a spike train (Figures 1B–1D and S1; 169.8±17 Hz, *n* = 11) and was typically expressed as a dense succession of multiple, temporally dispersed synaptic events maximally summating near the end of the stimulus and gradually waning over several seconds (Figure 1B–1D). These responses were reliably evoked over multiple applications of the same stimulation protocol (Figure 1C) and were completely abolished by bath application of 10 µM gabazine (GBZ; see below), indicating that they were GABA_A_-receptor mediated.

**Figure 2:**
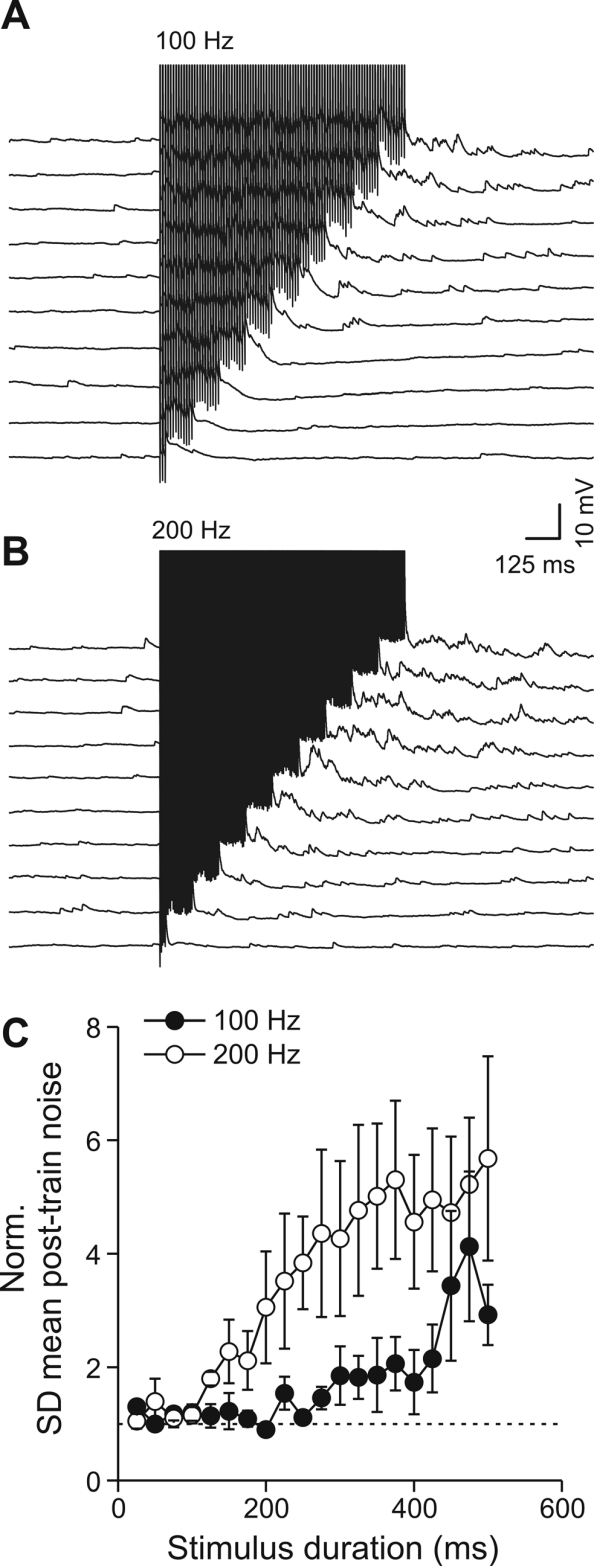
AP frequency and train duration required to induce delayed self-inhibition. (A and B) Current-clamp recordings from a FS interneuron showing increase of sIPSPs following directly evoked spike trains of various durations at frequencies of 100 (A) and 200 Hz (B). (C) Graph showing the relationship between the duration and frequency of trains and the average increase in delayed sIPSPs (*n* = 6). For this analysis, we used the SD of the mean membrane potential noise (a measure of asynchronous release) normalized to its pre-train level to quantify the change in spontaneous IPSPs (see Materials and Methods).

To characterize this effect in more detail, we examined the dependence of the delayed spontaneous inhibition on FS cell activity by changing the frequency and duration of train stimulations. We found that the amount of spike-train-induced increase in delayed inhibition was strongly dependent on both parameters (Figure 2), increasing as a function of FS train frequency and duration. We rarely detected delayed inhibitory events when FS interneurons fired APs at frequencies smaller than 100 Hz, despite a few exceptions at 60–70 Hz (data not shown), suggesting that this phenomenon requires relatively high frequency firing. At a stimulation rate of 100 Hz, the minimal train duration required to induce significant changes in post-train spontaneous synaptic events was 450 ms (normalized post-train noise = 3.4±1.3; ANOVA and post-hoc Dunnett's test, *p*<0.01, *n* = 6) whereas significant changes could already be observed with trains of 250 ms at 200 Hz (3.8±0.8; *p*<0.05). In general, the number of APs seemed to be the determining factor because with equal numbers of spikes, the post-train response was identical at all stimulation frequencies (see Figure S2). These results thus demonstrate a sustained and delayed GABAergic self-inhibition that is tightly coupled to the level of FS cell activity.

### The Delayed Self-Inhibition Is Mediated by Asynchronous Autaptic Release

Previous work has established that neocortical FS basket cells form several synapses with themselves (i.e., autapses [22],[34],[35],[46]). Indeed, it has been estimated using electron microscopy [34] and quantal analysis [46] that each FS interneuron can form between 12 and 22 autaptic contacts. This functional self-innervation supports fast single-spike-evoked synchronous GABA release and self-inhibition [22],[35],[36],[46]. The prominent and delayed increase in sIPSCs described above could be supported by self-inhibitory contacts onto FS cells. Alternatively, it may result from a more global potentiation of GABA release from nearby inhibitory neurons through a gap-junction coupling of activity or perhaps a retrograde signaling mechanism.

**Figure 3:**
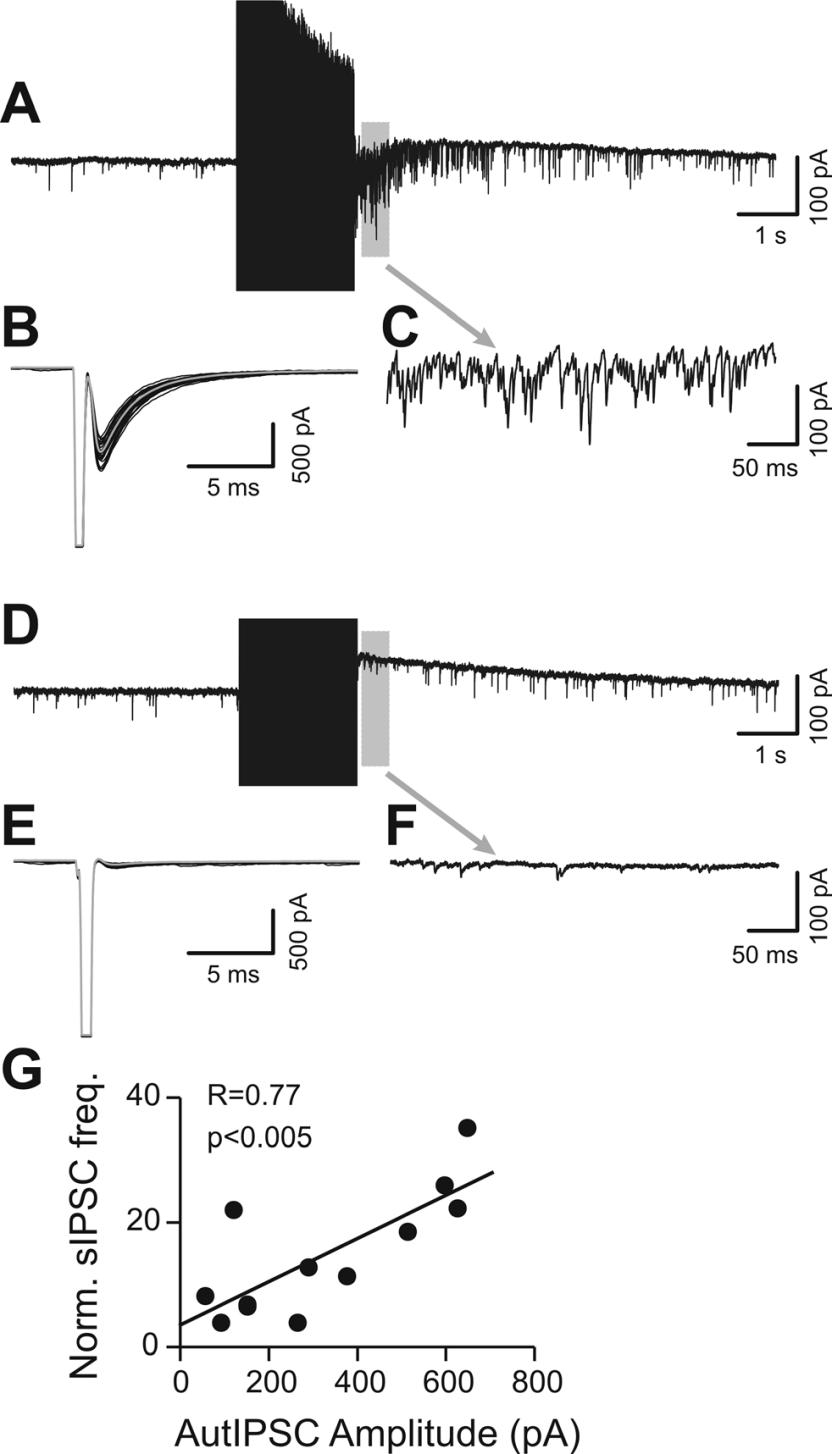
The delayed increase in sIPSC frequency is selectively expressed in autaptic FS interneurons. (A) Voltage-clamp recording from a FS cell, showing massive sIPSC activity following high-frequency voltage-clamp evoked APs (130 Hz, 2 s). (B) Voltage-clamp recording from the same neuron as in (A), showing prominent autaptic responses. Black traces are superimposed single-trial responses with average IPSC shown in gray. (C) Section of trace in (A) marked by gray bar, shown at an expanded time scale. (D–F) Voltage-clamp recording from another FS interneuron, showing the lack of increased spontaneous IPSCs following a similar AP train (gray-barred section expanded in [F]) and corresponding absence of autaptic response (E). (G) Plot representing the sIPSC frequency increase following AP trains versus autaptic size. Linear regression fit of the data showed a significant correlation (*n* = 11).

To distinguish between these possibilities, we first examined the incidence of FS-cell autaptic responses in parallel with the train-induced potentiation of sIPSC frequency. A delayed increase in spontaneous inhibition was observed in all FS interneurons showing autaptic transmission (Figure 3A–3C), whereas the rare cases (<10%) of FS cells with undetectable autaptic currents showed no increase of spontaneous events following AP trains (Figure 3D–3F). Moreover, the size of single-spike-induced autaptic currents was linearly correlated to spike-train-induced increases in sIPSC frequency (Figure 3G; *n* = 11). FS cells displaying robust increases of sIPSCs also showed strong synchronous autaptic responses. These results are consistent with the idea that autaptic terminals are a primary site of enhanced spontaneous GABA release after intense firing activity in FS interneurons.

**Figure 4:**
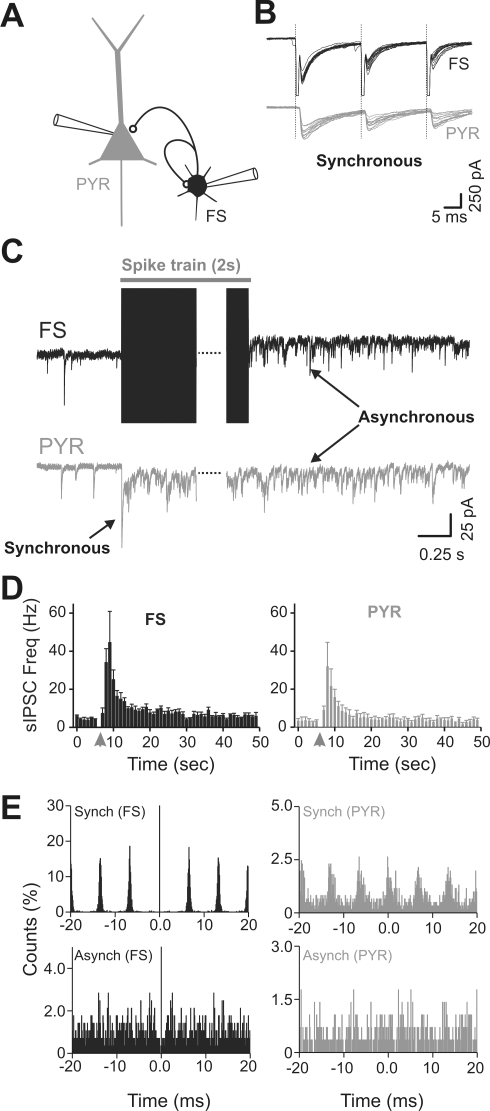
Paired recordings between autaptic FS interneurons and postsynaptic P cells reveal substantial and matching asynchronous transmission onto both target cells. (A) Scheme of the “equivalent” triple-patch recording configuration. PYR, P cell. (B) Voltage-clamp recordings of autaptic (black) and synaptic (gray) currents from a connected FS–P neuron pair, showing synchronized GABA-mediated responses to short voltage steps (vertical lines mark the onset of truncated spikes) elicited in the “presynaptic” FS cell. (C) High-frequency APs in the FS cell (2 s, 150 Hz) generate a massive increase in sIPSCs visible in both the FS and P cell recording. Note that spontaneous synaptic responses in the P neuron are sustained for as long as the autaptic ones after the stimulus train. (D) Plots of average sIPSC frequency before and after train (gray arrowhead) showed a similar time profile for FS (left panel) and P (right panel) cells (*n* = 4 pairs). (E) Top: Event-correlation histograms of autaptic (black, left) and synaptic (gray, right) IPSCs showing the synchronous nature of evoked release in both FS and P cells during high-frequency stimulation of the presynaptic FS cell. P neurons had a higher failure rate than FS cells, thereby explaining a lower count. Bottom: Correlograms of the high-frequency autaptic and synaptic events detected in the FS interneuron and P cell in the 2 s that followed spike trains show highly uncorrelated and asynchronous activity.

To more directly test whether the increase in sIPSC frequency originates from FS-cell autaptic terminals and not from retrograde synaptic signaling globally affecting both autaptic and extrinsic presynaptic terminals, we investigated the effect of FS-cell train stimulations in autaptically and synaptically connected pairs of FS and P neurons. This configuration (Figure 4A) is a virtual triple recording with one presynaptic and two postsynaptic cells: FS(pre)→FS (autaptic) and FS(pre)→P (synaptic). If AP firing in the FS cell triggers a similar and simultaneous increase of sIPSC frequency in both postsynaptic targets, then this phenomenon is likely due to purely presynaptic changes and not retrograde signaling to GABAergic terminals impinging on FS cells selectively.

FS and P cells were recorded using the same intracellular solution, resulting in inward GABA-mediated currents at a V_hold_ of −70 mV. Autaptic inhibitory postsynaptic currents (autIPSCs), evoked by voltage-clamp steps that initiated axonal APs in FS cells, were highly synchronized with postsynaptic responses, measured simultaneously in P neurons (unitary inhibitory postsynaptic current [IPSC] amplitudes: 211.4±38.6 and 115.9±28.5 pA in FS and P cells, respectively; *n* = 4; Figure 4B).

In the absence of stimulation, random unsynchronized sIPSCs occurred at similar, low frequencies in both FS and P cells (3.2±0.4 Hz and 2.2±0.3 Hz in FS and P neurons, respectively; *n* = 4). Presynaptic cell train stimulations produced a strong and consistent increase in the frequency of sIPSCs recorded in the self-innervated FS cell, which was mirrored in the postsynaptic P cell (Figure 4C and 4D). The time course of sIPSC frequency changes as well as their relative magnitude (decay time constant τ = 2.7±0.6 versus 4.2±1.5 s, and normalized sIPSC response measured at peak after train stimulations 21.2±10.1-fold versus 14.3±5.6-fold for FS versus P cell, respectively; Figure 4D) were not significantly different between FS and P neurons (paired *t*-test, *n* = 4).

We ran a cross-correlation analysis of both synchronous and asynchronous IPSCs in FS and P neurons. As expected, autaptic and synaptic events evoked during the spike train are highly correlated, as they result from synchronous release from the same presynaptic cell. In contrast, IPSCs in FS and P neurons occurring after spike trains were not correlated, indicating that although they originated from a common presynaptic FS cell they resulted from uncoordinated, asynchronous release of synaptic vesicles at individual synaptic terminals of the FS cell (Figure 4E). The occurrence of delayed asynchronous IPSCs in P neurons, which were not directly stimulated to fire APs, excludes the possibility of a retrograde signaling mechanism being responsible for this phenomenon. Our results instead indicate that the increase in spontaneous release originates from the terminals of the stimulated FS cell itself and that this change affects both autaptic and postsynaptic targets of the cell. The delayed and prolonged increase in sIPSC frequency in response to a spike train in FS interneurons could in principle result from spiking activity originating from nearby FS interneurons that would be activated by high-frequency firing of one FS cell. Since FS interneurons are known to have a relatively depolarized reversal potential for GABA-mediated responses [47],[48], summation of GABAergic events might lead connected FS interneurons to fire and thus transmit delayed synaptic activity onto the recorded neuron. Pair recordings between two closely spaced FS interneurons revealed that when one FS cell was stimulated with high-frequency trains in voltage-clamp mode, no spiking activity was ever detected in the second FS cell held in cell-attached mode, regardless of whether the two cells were connected (*n* = 4 and 12 for connected and unconnected pairs, respectively; Figure S3). This result indicates that the late increase of sIPSC frequency is not due to activity-dependent firing of other FS cells embedded in the network. Interestingly, when whole-cell configuration was established in the other FS interneuron previously held in cell-attached mode, a delayed high-frequency sIPSC activity was apparent in the postsynaptic FS cell (Figure S3), similar to that recorded in postsynaptic P neurons (Figure 4), indicating that asynchronous release is a common feature of FS-cell synaptic terminals, independent of postsynaptic target (autaptic, FS–FS, and FS–P). The delayed increase in sIPSC frequency in FS neurons is induced by spiking activity and yet is not precisely coupled to AP production. Moreover, it seems to promote vesicle fusion for periods outlasting the stimulus trains. It therefore may be described as a form of desynchronized or asynchronous quantal release [27].

### Ca^2+^ Dependency of Delayed Asynchronous Autaptic Transmission

The prevailing mechanism underlying asynchronous release is still a matter of debate, but it is generally thought to rely on at least two important factors: the accumulation of residual Ca^2+^ that builds up presynaptically after a train of APs and/or the high Ca^2+^ sensitivity of specific proteins of the synaptic vesicle release machinery, which may trigger fusion events even after [Ca^2+^]_i_ has dropped or when Ca^2+^ has diffused away from the active zone [26],[27],[49],[50].

To further determine the level of Ca^2+^ sensitivity of asynchronous release, we performed patch-clamp recordings from autaptic FS cells using internal solutions containing different Ca^2+^-buffering molecules. In these experimental conditions, the intracellular concentration of Ca^2+^ at autaptic terminals can be finely adjusted by internal perfusion of specific Ca^2+^ buffers while simultaneously monitoring the evoked (synchronous) or induced (asynchronous) GABA response in the same cell. The exchange and diffusion rates of the internal buffer solution from the cell soma region to the terminals allowed us to determine the time course of the effect, and thus make control and post-buffer observations in each cell at different time points following patch rupture.

**Figure 5:**
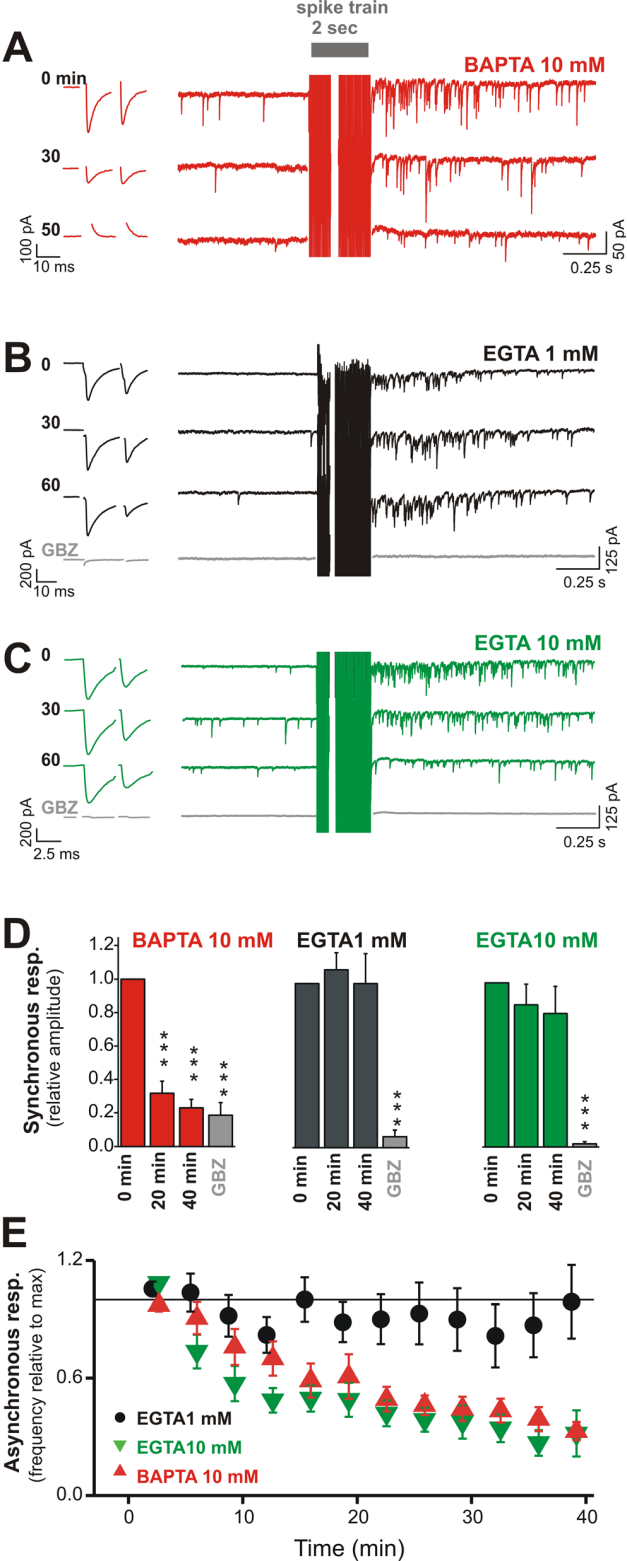
Synchronous and asynchronous autaptic release are differentially affected by fast- and slow-acting Ca^2+^ chelators. (A) Synchronous (left) and asynchronous (right) IPSCs recorded from autaptic FS cells immediately (*t* = 0) (top), 30 min (middle), and 50 min (bottom) after patch rupture with an intracellular solution containing 10 mM BAPTA. Note that both synchronous and asynchronous responses gradually disappear. (B) Synchronous and asynchronous IPSCs at three different time points (0, 30, and 60 min) while recording and loading the cell with a patch solution containing low-concentration (1 mM) EGTA. Neither the synchronous nor the asynchronous responses are affected by EGTA at this concentration. (C) Same as in (B) but with high-concentration EGTA (10 mM). In these conditions the fast single-spike-evoked IPSCs are stable for over an hour, but the asynchronous release is progressively blocked. In (A–C) (left) action currents (stimuli) were digitally removed to reveal synchronous autIPSCs. (D) Summary bar graph showing the normalized average synchronous autIPSC amplitudes in the presence of 10 mM BAPTA (red, *n* = 5), 1 mM EGTA (black, *n* = 5), and 10 mM EGTA (green, *n* = 7), at three time points (*t* = 0, 20, and 40 min) and after adding GBZ (10 µM in bath). Note that the synchronous response is blocked by BAPTA (***, *p*<0.001) at 20 and 40 min, but is preserved with both concentrations of EGTA. In these experiments, GBZ blocked all synchronous IPSCs (***, *p*<0.001). The small residual currents that were sometimes observed after addition of GBZ were clearly stimulation artifacts as they did not show any fluctuation. (E) Time course of normalized asynchronous autaptic release recorded from FS cells loaded with patch solutions containing 1 mM EGTA (black, *n* = 10), 10 mM EGTA (green, *n* = 8), or 10 mM BAPTA (red, *n* = 5). Note that both the 10 mM EGTA and 10 mM BAPTA intracellular solutions solidly reduce asynchronous release.

We first recorded from autaptic FS cells with an intracellular patch solution containing the fast-acting Ca^2+^ chelator BAPTA (10 mM). The amplitude of synchronous autIPSCs and degree of asynchronous release were monitored at regular intervals for as long as the holding current and access resistance were stable. As previously shown [22], intracellular BAPTA almost completely blocked synchronous autaptic transmission. The peak autIPSC amplitude (−205±73 pA, *n* = 5), was reduced by 68.3%±7.2% and 77.1%±5% when measured 20 and 40 min after the initial response, respectively (*n* = 5, *p*<0.001, paired *t*-test). Moreover, intracellular diffusion of BAPTA also progressively suppressed asynchronous release from autaptic terminals (Figure 5E; post-train sIPSC frequencies at times 20 and 40 min were 49%±6% and 33%±5% of initial response; *n* = 12 and 10, respectively, *p*<0.001 in both cases, paired *t*-test). This result indicates that the delayed increase in sIPSC response depends on Ca^2+^ levels in autaptic terminals. Synchronous and asynchronous release were equally and rapidly blocked by the addition of the broad Ca^2+^-channel blocker cadmium (Cd^2+^, 100 µM) in the bath perfusion (data not shown), indicating that both release modes are Ca^2+^ dependent.

We then examined the effects of the relatively slow Ca^2+^ buffer EGTA on synchronous and asynchronous autaptic release [29],[51],[52]. We reasoned that if asynchronous autaptic release depends on a more sustained Ca^2+^ increase than synchronous release, it should be specifically affected by a high concentration of EGTA, which, due to its slow binding kinetics, would not affect early transient Ca^2+^ increases but would buffer later, more prolonged transients [50],[53]. To make sure that the lengthy whole-cell recording and intracellular dialysis of the cell were not in themselves affecting the response, we first tested the effect of EGTA at a low concentration. At 1 mM, EGTA had no effect on either synchronous or asynchronous release. Synchronous and asynchronous GABAergic responses were stable for several tens of minutes and over an hour in some cases (Figure 5B, 5D, and 5E; peak synchronous autIPSC amplitude  = 108%±10% and 100%±18%, and post-train increase of sIPSC frequency  = 90%±12% and 99%±19%, for 20 and 40 min after whole-cell breakin, respectively). Values were not significantly different from control at time 0. In contrast, when FS cells were recorded with 10 mM EGTA, the asynchronous response was significantly reduced (Figure 5E; 41.9%±6.6% and 23.7%±10.1% at 20 and 40 min, respectively; *n* = 8 in both cases, *p*<0.001, paired *t*-test), whereas synchronous release was stable (peak autIPSC amplitude  = 87%±13% and 81%±17% at 20 and 40 min, respectively; *n* = 7 and 6, not significant). At this higher EGTA concentration, asynchronous responses were also significantly reduced compared to control values obtained with 1 mM EGTA at time points 20 and 40 min (*p*<0.001, ANOVA Bonferroni's post-hoc analysis) but did not differ from values observed in the presence of 10 mM BAPTA. Note that the initial peak amplitude of synchronous autIPSCs (−213±89 pA, −316±97 pA, and −384±132 pA for BAPTA 10 mM, EGTA 1 mM, and EGTA 10 mM, respectively; *n* = 5, 10, and 7) as well as the initial magnitude of train-induced sIPSC frequency increase (22.6±8.6-fold, 24.1±10.5-fold, and 23.8±7.4-fold increase; *n* = 8, 8, and 13, respectively) did not differ between the three groups (ANOVA Bonferroni's post-hoc test).

**Figure 6:**
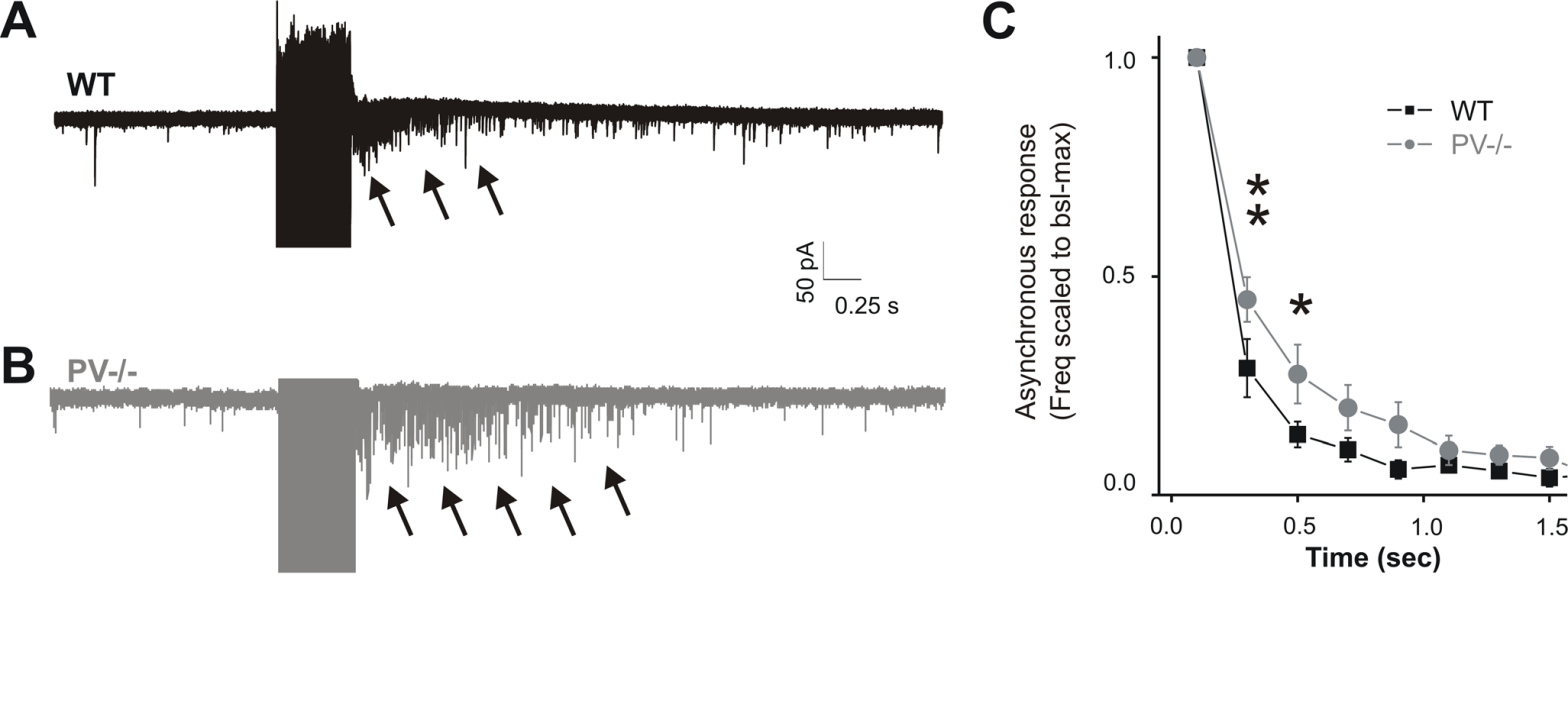
Lack of endogenous PV enhances asynchronous release from FS interneuron autaptic terminals. (A) Representative traces (overlay of 20 consecutive sweeps from one experiment) showing train-induced asynchronous autaptic release (denoted by arrows) from a typical WT mouse FS cell. (B) The same stimulus train (500 ms, 200 Hz) in a FS cell from a PV^−/−^ mouse generates a more sustained asynchronous response with slower decay. (C) Graph summarizing the averaged increase of asynchronous release against time in WT and PV^−/−^ mice (*n* = 8 for both groups). The sIPSC frequency was calculated in bins of 200 ms and normalized to the maximum response recorded immediately after the end of the spike train (*t* = 0). The result of a two-way ANOVA indicated that both “time” and “mouse strain” have a significant effect on the asynchronous response (*p*<0.001). Furthermore, Bonferroni's post-test revealed that PV^−/−^ values were significantly different from WT values during the first two time bins after the normalized maximum (*p*<0.01 and *p*<0.05, respectively).

To test whether reducing endogenous Ca^2+^-buffering capacities by FS interneurons affects asynchronous release, we performed whole-cell recordings from FS cortical interneurons in slices from control wild-type (WT) and PV null mice (PV^−/−^) to examine whether PV is involved in modulating the asynchronous response. These experiments were performed using an internal solution containing 0.05 mM EGTA, a concentration that should not interfere with asynchronous release, and should not rescue PV-mediated Ca^2+^ buffering, which was estimated to be equivalent to that of approximately 1 mM EGTA in cerebellar interneurons [54]. Peak amplitudes of single-spike-evoked unitary autIPSCs recorded in PV^−/−^ mice were not significantly different from those obtained from WT (−251.1±34.6 versus −281.1±37.5 pA, WT versus PV^−/−^, respectively; *p*>0.05, independent *t-*test, *n* = 8 cells for both groups). In response to high-frequency stimulations (500-ms train, 200 Hz), the decay of activity-dependent asynchronous release was significantly slower in FS cells from PV^−/−^ than from WT mice, particularly in the 500-ms time window following presynaptic AP trains (Figure 6; normalized IPSC frequency for WT versus PV^−/−^, 29%±7% versus 45%±5% at time 0.2–0.4 s and 14%±3% versus 28%±7% at time 0.4–0.6 s; ANOVA post-hoc comparisons, *p*<0.01 and *p*<0.05, respectively; τ = 0.18±0.01 versus 0.31±0.03 s, WT versus PV^−/−^, respectively). The change in duration of asynchronous release between WT and PV^−/−^ was not accompanied by a difference in asynchronous release magnitude calculated 100 ms after the spike trains (*p*>0.05, independent *t-*test, data not shown).

Altogether, these results indicate that asynchronous autaptic release depends more on intra-terminal Ca^2+^ build-up than single-spike-induced synchronous release.

Importantly, asynchronous autaptic release of GABA from FS interneurons was present in perforated-patch experiments using gramicidin, which leaves intracellular ion homeostasis unperturbed (data not shown; *n* = 2 out of 4 FS cells), ruling out the possibility that asynchronous release is due to intracellular dialysis of endogenous molecules and/or Ca^2+^ buffers.

### FS Interneurons Self-Regulate Their Own Input–Output Response to Physiologically Relevant Stimuli through Autaptic Asynchronous Release

**Figure 7:**
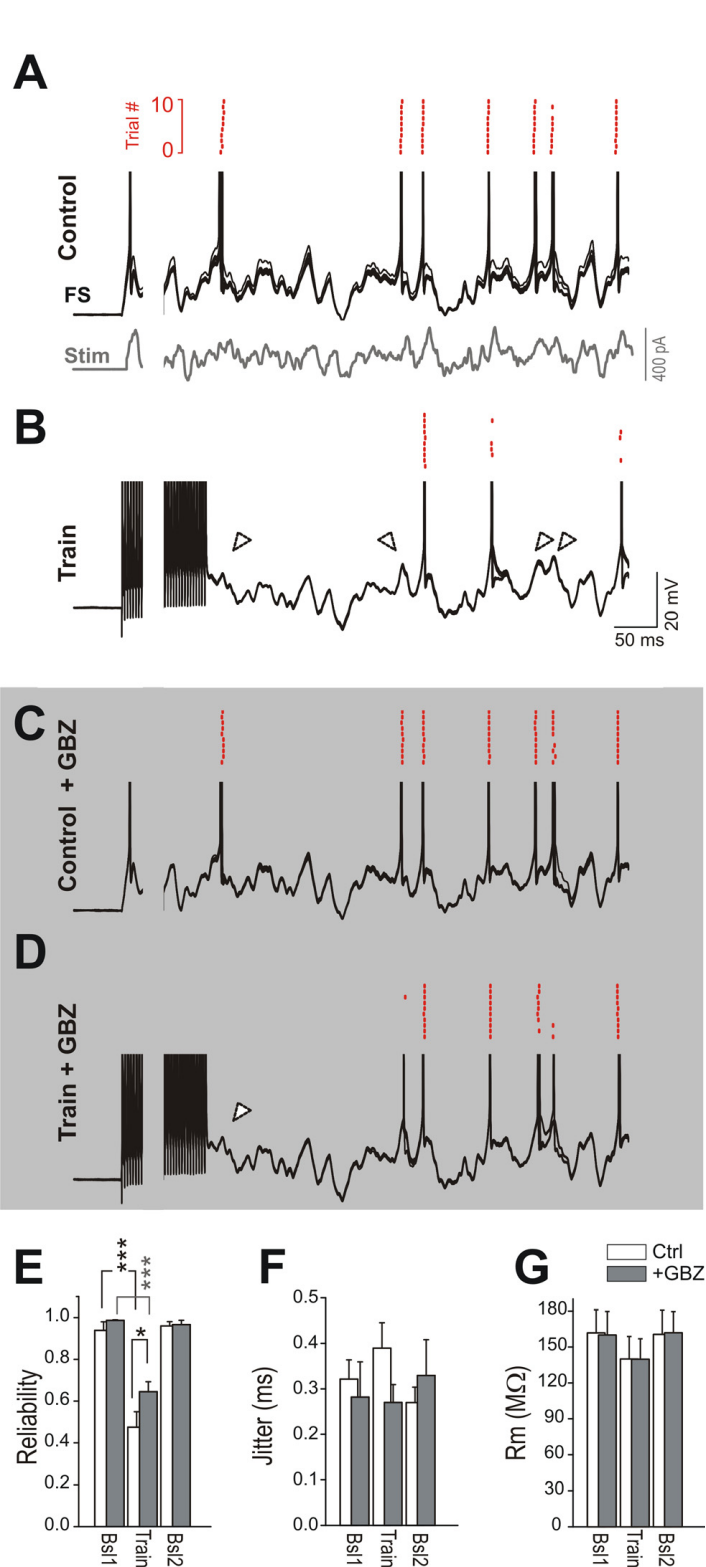
Self-modulation of FS interneuron firing by asynchronous autaptic GABA release. (A) Membrane potential response and spiking activity of a FS interneuron (black traces, ten superimposed consecutive trials) generated by the repeated injection of in-vivo-like frozen noise stimulations (gray). Raster plot of spike times over ten trials are shown in the red inset on top. Note that spikes are evoked reliably and with high precision over repeated trials. The injected current waveform is only shown for (A). (B) Response of the neuron of (A) to the same stimulation (ten trials) but in which the first half of the frozen noise waveform was replaced with a high-frequency (300 Hz) train of brief current steps that each evoked a single AP. Note that many of the spikes, which were reliably evoked during the control stimulations, are now completely missing from the trace (white arrowheads) and raster plot (red dots) in the post-train period. (C) Response of the same FS cell as in (A) to the control stimulation in the presence of GBZ (10 µM, applied by local perfusion). In this condition, the firing pattern response is indistinguishable from that shown in (A). (D) Response to stimulus protocol as in (B), but in the presence of GBZ. The GABA_A_-receptor antagonist restores FS-cell reliability. In (A–D) APs were truncated and a portion of the first 500 ms was digitally removed for display purposes. (E) Summary graph of six FS cells showing spike reliability with and without asynchronous-release-inducing spike trains (train and controls, Bsl1 and Bsl2, respectively) in the absence and presence of GBZ (white and gray bars, respectively). ***, *p*<0.001. The single asterisk marks statistical difference between post-train reliability values in the control and GBZ conditions (*p*<0.05). (F) Summary plot of jitter (SD of spike timing calculated over ∼20 stimulations) with and without spike trains in the absence and presence of GBZ (white and gray bars, respectively). (G) Average membrane resistance (*R*_m_) before and after spike trains in the absence and presence of GBZ (white and gray bars, respectively), suggesting lack of tonic GABA-mediated conductance when GBZ was applied through a local perfusion system. Spike reliability and precision were calculated within a single time window of 500 ms post train.

The experiments described above show that high-frequency activation of FS neurons prolongs GABAergic self-inhibition for several seconds after the stimuli. This suggests that FS cell properties may be modulated by their own output, long after spiking activity has ended. In particular, delayed asynchronous release could modulate the ability of neurons to reliably encode a particular stimulus input into a specific spike pattern. To test this hypothesis, FS interneurons were recorded with a physiological intracellular chloride concentration yielding an equilibrium potential for GABA_A_-mediated responses similar to that previously recorded in perforated-patch experiments (E_Cl_ = −52 mV; see Materials and Methods; [47],[48]). FS cells recorded in current-clamp were stimulated by injecting in-vivo*-*like and synaptic-like noisy current waveforms [3],[17]. We assessed the reliability and precision of firing of recorded cells by repeatedly (10–20 times) applying the same stimulus waveform (frozen noise stimulations) and evaluating the consistency of the output spike sequence. Similar to previously published results [3], this type of stimulus generated a consistent trial-to-trial firing pattern, in which APs occurred reliably and with sub-millisecond precision across trials (Figure 7A, 7E, and 7F) in all six recorded FS interneurons (reliability = 94.9%±4.1%; jitter = 0.32±0.04 ms; average firing rate = 27±3 Hz). To test the effect of asynchronous release on this precise and reliable firing, the first portion of the stimulus waveforms was replaced by high-frequency current pulses (300 Hz, 500 ms) to generate high-frequency spike trains in FS cells. Spike trains were followed by a period in which AP firing was less reliable in response to synaptic noise stimulations (spike reliability reduced from 94.9%±4.1% in control to 48.0%±7.5% after train stimulation; *p*<0.001, paired *t*-test), whereas spike timing precision did not show significant change (jitter = 0.39±0.06 ms; Figure 7B, 7E, and 7F). Importantly, when we returned to the control condition, injecting only the frozen noise, the reliability and jitter returned to pre-train levels (97.1%±2% and 0.27±0.03 ms, respectively), indicating that changes in spike reliability were not due to nonspecific alterations of firing patterns induced by drifts of recording conditions.

Changes of FS-cell spike reliability could be due either to asynchronous autaptic release or to a train-induced shunt mediated by a slow Ca^2+^- or Na^+^-dependent, K^+^-channel-mediated after-hyperpolarization (AHP; for reviews, see [55],[56]). Indeed, although the cell resistance did not significantly decrease in the interval between stimulation periods compared to the before and after control conditions (162±19 versus 140±19 versus 161±20 MΩ; *p*>0.05; Figure 7G), a prominent hyperpolarization was always present immediately after the spike trains.

To directly test the actual contribution of asynchronous GABA release to the spiking reliability reduction seen in FS cells, we repeated the frozen noise stimulations in the presence of 10 µM GBZ and compared the response of FS cells before and after blocking GABA_A_-receptor-mediated transmission. In the presence of GBZ, AP trains still had a hampering effect on the reliability of FS firing (Figure 6D and 6E; 65.2%±4.8% versus 99.8%±2.3%; *p*<0.001, paired *t*-test), confirming a shunt by AHP-dependent mechanisms, presumably K^+^ channels. However, the effect was significantly smaller than in the absence of GBZ, suggesting that delayed asynchronous autaptic release played a role in reducing spike reliability in FS interneurons (Figure 7E; 46.9% versus 34.6% reduction in control versus GBZ; *p*<0.05). Thus, although the dominant effect of the train on firing was attributable to intrinsic shunting currents, our results indicate that approximately 26% [(46.9%−34.6%)/46.9%] of the drop in spike reliability was due to GABAergic autaptic asynchronous release. Again in GBZ recordings, nonspecific drifts of recording conditions were ruled out, as repeated control experiments performed after delivering the high-frequency trains yielded values for spike reliability that were indistinguishable from those obtained in the first control sequence. Importantly, these experiments were performed using a local perfusion system that efficiently removed ambient GABA, as evidenced by the lack of significant change in the total FS-cell membrane resistance when switching the flow from control to GBZ, and back to control solution (Figure 7G; 160±20 MΩ versus 140±17 MΩ versus 162±18 MΩ; *p*>0.05), ruling out a role for tonic inhibition in these effects. Values of membrane resistance (*R*_m_) in the presence of local perfusion are higher than those previously reported for FS interneurons [21],[36],[38]. However, *R*_m_ values for FS cells were in line with previously published results in all other experiments that did not use a local perfusion (103.5±4.5 MΩ, range 55–195 MΩ, *n* = 36). In addition, there were no appreciable differences in asynchronous release in experiments with or without local perfusion, ruling out the possibility that asynchronous release was due to increased impact of small GABAergic events.

### Control of P-Cell Firing by Asynchronous Release from FS Interneurons

In P neurons, GABAergic contacts from FS cells are mostly located at the soma, contributing to the electrotonic shunt responsible for synchronization during rhythmic cortical activity. We have shown that asynchronous release from FS cells affects both the FS cells via autapses and the postsynaptic P cells (Figure 4C). We therefore tested whether the effect of this prolonged and desynchronized inhibition could change the input–output properties of P neurons in response to frozen synaptic noise stimulations. In these experiments P neurons did not fire high-frequency trains of APs, so that the effect of desynchronized inhibition from FS interneurons could be tested more accurately without any accompanying slow AHP mediated by K^+^ channels.

**Figure 8:**
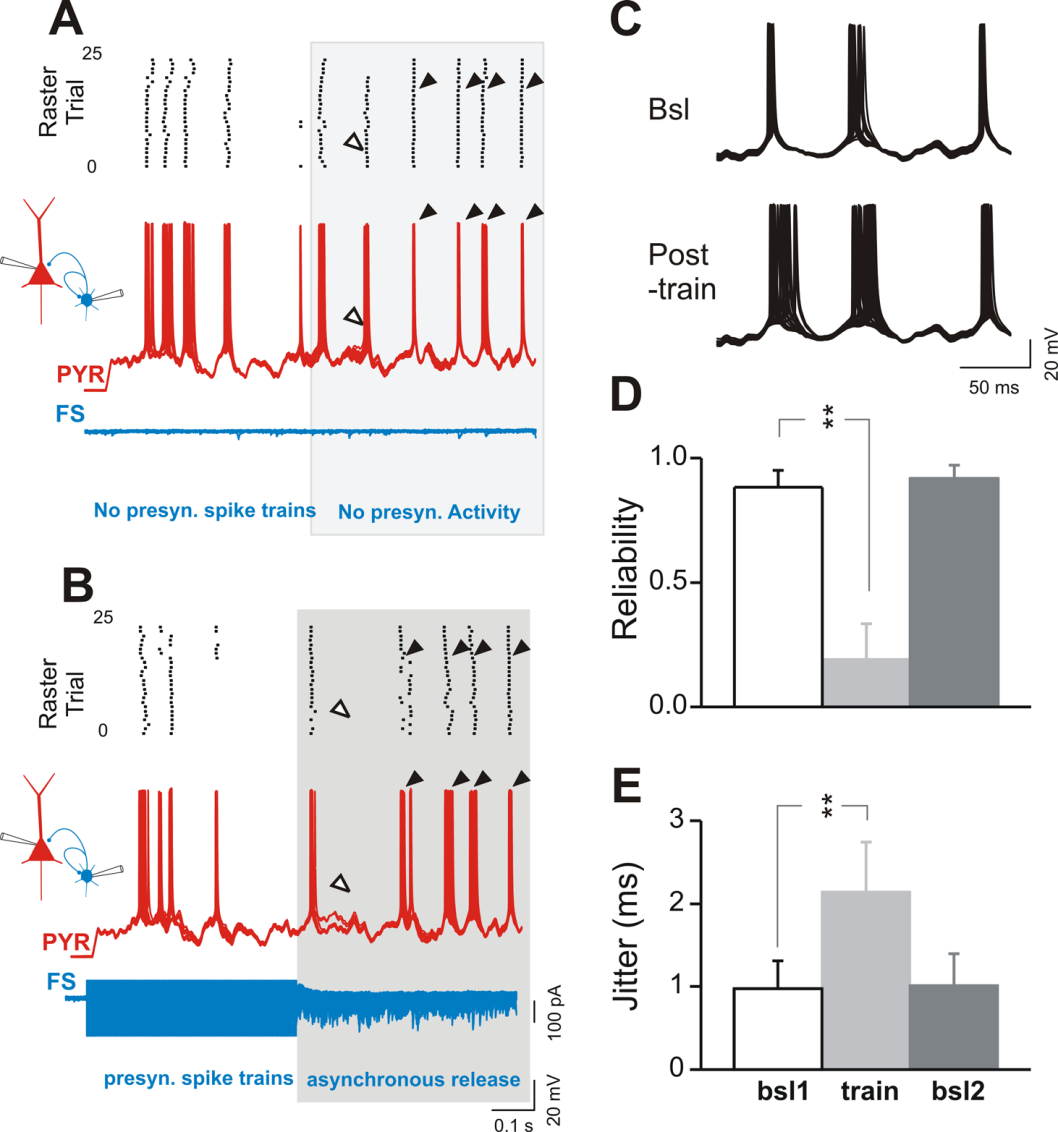
Asynchronous synaptic release of GABA deteriorates the overall precision and reliability of APs in P neurons. (A and B) The scheme on the left refers to the paired-recording configuration. One presynaptic FS interneuron is synaptically connected to itself and to a postsynaptic P neuron (PYR). Representative current-clamp overlapped traces recorded from a P neuron (red traces) stimulated with frozen noise current injections (30 identical sweeps) in the absence (A) and presence (B) of a spike train (300 Hz, 500 ms) elicited in a presynaptic FS interneuron. The blue trace is the voltage-clamp recording of the presynaptic FS cell. Note that spike trains in the FS interneuron induce asynchronous release (B). P-neuron spike precision and reliability deteriorates both during and over 500 ms after the train (gray box) during asynchronous synaptic release. Upper panels show spike raster plots collected from the same experiment. Each dot represents an AP. Black arrowheads point to examples of spike clusters (single APs evoked upon repeated presentations of the stimulus within a relatively narrow time window; see Materials and Methods) showing increased jitter after the presynaptic stimulus trains. White arrowheads point to spikes that disappeared because of asynchronous release of GABA. (C) Same recording of cells in (A) (control; Bsl) and (B) (post-train): the last three spike clusters (all 25 superimposed traces) are shown with a higher temporal resolution to highlight the dramatic increase in jitter during asynchronous GABAergic activity. (D and E) Average plots of spike reliability (D) and precision (E) of P neurons in control condition (frozen noise in the absence of presynaptic FS cell activity; baseline 1 [bsl1]; white columns), in the test condition (presence of asynchronous release; train; light gray columns), and in a subsequent control (baseline 2 [bsl2]; dark gray columns). The second control was recorded after the asynchronous release sequence to test for the recovery of both parameters. Spike reliability and jitter are shown for time intervals 0–200 and 200–300 ms, respectively, after the high-frequency train evoked in presynaptic FS cells. It was not possible to calculate jitter within the first 200 ms following presynaptic stimulation, because of lack of spikes as a consequence of decreased reliability. **, *p*<0.01; *n* = 4 in (D) and *n* = 5 in (E).

To investigate the effect of FS asynchronous synaptic release on P cell firing, we performed paired recordings between connected FS and P neurons. P neurons were patched with an intracellular solution resulting in a Cl^–^ reversal potential close to the physiological level reported for cortical P cells (see Materials and Methods; [47]). FS interneurons were loaded with a high-chloride intracellular solution to monitor the instantaneous dynamics of asynchronous autaptic release. Repeated noisy current stimulations were injected in P neurons in the presence or absence of presynaptic spike trains (Figure 8). To analyze the effect of FS asynchronous release, we focused on the 500-ms period after presynaptic spike trains.

P-cell firing responses to frozen noise current injections were very precise with low failure rates (Figure 8A), as previously reported [3]. Train stimulations of the presynaptic FS cell induced delayed asynchronous release that was accompanied by a change in the firing pattern of the P neuron both during and after the train in the FS cell (Figure 8B). When asynchronous-inducing trains were generated in FS–P neuron pairs, the reliability and precision of firing significantly deteriorated (>3-fold decrease in reliability within the first 200 ms following presynaptic trains in FS cells; 88.4%±6.7%, 18.9%±14.5%, and 91.9%±5.2% for control 1, train, and control 2, respectively; *p*<0.001, ANOVA control 1 versus train reliability; *p*<0.01, post-hoc Bonferroni test, *n* = 4; Figure 8D) (∼2-fold increase in spike jitter between 200 and 300 ms following presynaptic trains in FS cells; 1.0±0.3, 2.1±0.6, and 1.0±0.4 ms for control 1, train, and control 2, respectively; *p*<0.01, ANOVA control 1 versus post-train jitter; *p*<0.01, post-hoc Bonferroni test, *n* = 5; Figure 8C and 8E). Similarly to the case of autaptic FS cells alone (Figure 7), changes of P-neuron spike precision and reliability could not be accounted for by nonspecific drifts of recording conditions, as evidenced by identical spike jitter and reliability obtained in control experiments (without FS-cell spiking) performed before and after inducing asynchronous release by presynaptic spike trains (*p*>0.1). The effect of asynchronous release on spike reliability and precision in P neurons was greater in the first 200 and 300 ms following presynaptic spike trains, respectively, and recovered within a few hundred milliseconds later (data not shown). Interestingly, the immediate large effect of asynchronous release on P neurons was reduced spike reliability. Later, during the decaying phase of asynchronous release, spikes could be evoked more reliably, but with a much reduced precision (Figure 8D and 8E). These results suggest that the asynchronous inhibitory inputs from FS cells can interfere with the reliable and precise spiking of P cells.

## Discussion

The results of these experiments indicate that PV-positive FS basket cells of the neocortex respond to high-frequency firing with a massive and prolonged self-inhibition mediated by asynchronous autaptic release. This alternate mode of release is regulated by intra-terminal [Ca^2+^] differently than synchronous GABA release. Functionally, asynchronous release of GABA results in a delayed inhibition lasting for several hundreds of milliseconds, which reduces spike reliability of FS cell onto themselves, as well as spike reliability and precision of postsynaptic P neurons.

### A Delayed Increase in Self-Inhibition in FS Interneurons Mediated by Autaptic Asynchronous Release

We observed that when cortical FS interneurons are stimulated with trains of spikes at high frequencies, a massive increase of GABA-mediated events follows the stimulus train for several seconds. Theoretically, this self-induced delayed increase in sIPSC frequency observed in FS cells could result from at least three mechanisms: (1) polysynaptic activation\recruitment of other GABAergic cells within the circuit, (2) retrograde signaling to presynaptic GABAergic terminals, or (3) asynchronous GABA release from FS-cell autaptic terminals.

Polysynaptic activation would imply that stimulation of a FS neuron is capable of triggering spikes in some synaptically connected cells, which would then activate other interneurons, leading to a feedback inhibition. This type of mechanism has been described to explain delayed disynaptic inhibition between P cells [14],[57],[58]. However, disynaptic inhibition between cortical neurons depends on glutamatergic transmission [57],[58], and all the experiments described herein were performed in the presence of glutamate receptor antagonists. Moreover, network-triggered, polysynaptic self-inhibition is unlikely between GABAergic FS interneurons. This was confirmed by direct experiments, in which spike trains evoked in FS interneurons failed to elicit firing in neighboring FS cells recorded in cell-attached mode, i.e., with unperturbed intracellular chloride homeostasis (Figure S3). This result excludes the possibility that a relatively depolarized reversal potential for GABA-mediated responses in FS cells [47],[58] might summate and cause other interneurons to fire.

As the increase in sIPSC frequency follows high-frequency trains of spikes, it is possible that this intense activity triggers the activation of a retrograde signaling pathway increasing GABA release from presynaptic neurons. A similar, albeit more persistent, phenomenon described in the cerebellum was shown to involve postsynaptically released glutamate acting on presynaptic NMDA glutamate receptors [59]. Retrograde release of glutamate [60] or endocannabinoids (reviewed in [61]) is evoked by elevations of somatodendritic [Ca^2+^], which rapidly trigger the synthesis or release of the retrograde molecule. This rapid mode of action is incompatible with our observation that high concentrations of either BAPTA or EGTA in the recording pipette blocked delayed self-inhibition in FS cells slowly (tens of minutes), which is inconsistent with a rapid somatodendritic diffusion of these compounds. The time course of this effect is more consistent with the diffusion of these compounds to presynaptic terminals. Indeed, the time required for BAPTA to block delayed inhibition closely matches the time course of its effect on synchronous autaptic responses. This concurrence in the time course of the two effects indicates that both mechanisms are located remotely at the same autaptic sites. Moreover, the time course of the BAPTA effect in our experiments was similar to the one previously observed with connected FS–P cell pairs and is consistent with simulations estimating the diffusion speed of internally infused Ca^2+^ chelators from the soma to presynaptic terminals [51].

The delayed increase of inhibition in response to spike trains cannot be accounted for by electrical coupling. Indeed, gap-junction coupling functions as a low-pass filter: ∼100-mV spikes are sensed as ∼1-mV spikelets in electrically coupled cells [62],[63]. It is therefore unlikely that spikelets might summate in secondary FS cells to produce firing and GABA release that would result in the asynchronous responses reported here. This is further supported by the described lack of firing in postsynaptic FS cells. Moreover, the differential blockade of synchronous versus asynchronous release with 10 mM intracellular EGTA is inconsistent with asynchronous release generated in one or more electrically coupled interneurons.

Two other lines of evidence support the hypothesis that the delayed sIPSCs result from asynchronous autaptic release. First, this phenomenon occurred only in FS cells showing functional autaptic transmission, and the size of the autaptic response correlated with the increase of delayed sIPSCs. Secondly, asynchronous GABAergic events following a spike train in FS interneurons were also observed in connected P neurons and other postsynaptic FS interneurons, which did not fire APs and were held at hyperpolarized potentials. This increase of GABAergic events in P cells was very similar in amplitude and time course to that observed in parallel in FS cells. Altogether, these results indicate that retrograde signaling from FS cells to presynaptic GABAergic terminals can be ruled out. We therefore conclude that intense spike trains in FS interneurons induce delayed and prolonged asynchronous GABA release onto themselves and other postsynaptic targets.

### Properties of FS Asynchronous Release

Our results show that asynchronous autaptic release from FS interneurons is large (>10-fold increase in sIPSC rate) and could be reliably induced for as long as the cell integrity was maintained. Most of our recordings were made in preparations from animals at an age (18–25 d old) where neuronal maturation of the FS cell connectivity pattern is well advanced [64],[65], Moreover, asynchronous release was also observed in occasional experiments performed in slices obtained from mature animals (>100 d old; data not shown), indicating that this phenomenon is not limited to a narrow developmental period.

Asynchronous autaptic release required trains of spikes at relatively high frequencies (>100 Hz). Although this is consistent with stimulation protocols used to evoke asynchronous release in many other studies [27], this substantially differs from hippocampal CCK-positive interneurons, which show asynchronous GABA release in response to lower presynaptic spike frequencies (50 Hz, trains of ten spikes; [20]). As pointed out earlier, cortical GABAergic cell subtypes fire at different rates (and with different patterns) when depolarized by long current injections [9],[10]. CCK-positive cells typically display an adapting firing pattern; they cannot sustain high-frequency firing rates (>50 Hz; [39],[68]) and can only be recruited transiently by repetitive stimulation [66]. In contrast, neocortical FS interneurons can sustain very high frequency firing when stimulated both in vitro [21],[36]–[39] and in vivo [40],[41],[67]. In anesthetized or awake animals FS cells have been reported to fire high-frequency trains of APs that reliably match cortical ripples (up to 200 Hz, >100 ms; e.g., Figure 43 of [68]) and very fast oscillations (400–600 Hz) induced by sensory stimulations [44],[45]. Moreover, FS cells can fire >100 Hz for several hundreds of milliseconds in vivo in response to extracellular synaptic stimulations [43],[69]. Although these periods of very high- frequency firing in vivo are in general briefer than the trains used here to characterize the response, preparations of reduced complexity such as acute brain slices can be deprived of several metabolic precursors and lack the constant excitatory drive as well as neuromodulation originating from subcortical nuclei. As a consequence, neurons in brain slices are more quiescent than in vivo and may thus require more sustained stimulations. Notably, in pathological conditions such as during epileptic discharges in vivo, FS interneurons were found to fire at very high frequencies (up to 800 Hz) for hundreds of milliseconds [70],[71]. Nevertheless, our results show that asynchronous autaptic release is evident when FS interneurons fire trains of APs at 100 Hz for a few hundreds of milliseconds, and becomes more prominent at higher, physiologically relevant frequencies.

### The Differential Ca^2+^ Dependance of Synchronous and Asynchronous Release

Given the prevailing view that transmitter release at PV-positive basket cell terminals is rapid, highly efficient, and temporally precise [25], our finding of a prominent asynchronous release component at FS cell autapses and synapses was rather unexpected. The observation that fast synchronous autaptic release is progressively blocked by intracellular infusion of BAPTA, but insensitive to the slow Ca^2+^-buffering action of EGTA, is fully consistent with published data indicating that temporal precision of hippocampal basket PV-positive interneurons may be enhanced by the tight coupling of P/Q-type Ca^2+^ channels and Ca^2+^ sensors that trigger vesicle release at their active zones [20],[51]. This mechanism is also likely present in neocortical FS cell terminals, which were found to express the same type of presynaptic Ca^2+^ channels [72].

In contrast to synchronous release, which was unaffected by the slow Ca^2+^ buffer EGTA, even at 10 mM, the delayed and long-lasting asynchronous autaptic release was blocked by high concentrations of EGTA. As suggested previously for hippocampal CCK-positive basket cells, the effect of the slow Ca^2+^ buffer may result from a reduction of the long-lasting presynaptic Ca^2+^ transient following repeated stimulations [20]. The difference in asynchronous release between hippocampal PV-positive and CCK-positive basket neurons following trains of APs at low frequencies (50 Hz) was attributed to the expression by the latter of relatively slow N-type presynaptic Ca^2+^ channels, which are more loosely coupled to exocytosis [20]. It is known that both hippocampal [20],[51] and neocortical [72] FS cells express P/Q-type Ca^2+^ channels, which tightly couple Ca^2+^ entry and synaptic vesicle exocytosis at their terminals. However FS cells can fire at rates at least one order of magnitude higher than the low-range cutoff frequency (>50 Hz), and therefore these neurons are more likely to induce long-lasting residual Ca^2+^ signals at their terminals. The results of our experiments indicate that residual Ca^2+^ plays a major role in asynchronous release. Whether the two modes of release (synchronous and asynchronous) involve the same vesicle pools, release from the same active zones, and/or depend on the same Ca^2+^-sensitive elements of synaptic vesicle release machinery is yet to be clarified.

Consistent with this model, we found that the effect of deleting the endogenous Ca^2+^ buffer PV in PV^−\−^ mice was a small, but significant, increase in autaptic asynchronous release from FS autaptic terminals, suggesting a role of PV in limiting the buildup of residual Ca^2+^ (see Figure 7 of [53]). Similarly, in PGC-1α^−\−^ mice characterized by decreased PV levels in hippocampal interneurons, asynchronous release onto dentate gyrus granule cells during and after 40-Hz train stimulations (500 ms) was significantly larger than in WT animals [73]. Thus, at synapses between PV-positive interneurons and hippocampal granule cells, asynchronous release is already observed at lower frequencies (40 Hz) and is clearly dependent on PV levels in perisomatic inhibitory terminals. However, previous work using a similar approach showed that asynchronous GABA release at cerebellar interneuron terminals was decreased in slices from PV^−/−^ mice [52]. The discrepancy between this and our study could be accounted for by different developmental stages analyzed, different brain areas and cell types, and/or different expression of presynaptic molecular players involved in determining the sensitivity of synaptic vesicle release. Further experiments are required (including perhaps presynaptic calcium imaging) to clearly assign a role of PV in synchronous and asynchronous release originating from cortical FS interneurons.

The specific sensitivity of asynchronous release to high concentrations of EGTA, together with its modulation by the endogenous Ca^2+^ buffer PV, raises the question of whether it results from dialysis of endogenous buffers and/or other important metabolites that are lost during invasive whole-cell recordings. It is noteworthy that large and robust asynchronous release (both autaptic and synaptic) was always detectable immediately after patch rupture (<1 min) in all of our whole-cell experiments. A presynaptic washout of metabolic compounds in the intra-terminal cytoplasm during the first minutes of whole-cell recording is highly unlikely, as indicated by the timing (tens of minutes) required for 10 mM BAPTA and 10 mM EGTA to reach presynaptic sites and block asynchronous release. Moreover, our experiments in the presence of 1 mM EGTA in the whole-cell pipette showed that the magnitude of asynchronous release remained constant for long recordings (>50 min) in all tested neurons. Possible effects related to whole-cell dialysis in the first minutes of recordings would have resulted in changes of asynchronous release properties soon after whole-cell establishment. Finally, asynchronous release was detectable in few perforated-patch experiments (data not shown), directly ruling out the possibility that it is due to imbalanced ion homeostasis and/or dialysis due to whole-cell recording.

### Functional Role of Autaptic and Synaptic Asynchronous GABA Release from FS Interneurons

FS interneurons are fast and precise elements of cortical circuits, instrumental in synchronizing large populations of neurons in a network ([8]; reviewed in [42],[74]). Indeed, we previously found that large synchronous, single-spike-dependent autaptic release of GABA dictates the timing of AP firing in FS cells, with potential implications for keeping these neurons synchronized during network activity [22],[35].

Our results suggest that the function of FS cells may not be limited to the phasic nature of fast cortical inhibition but might, under some conditions, be involved in more sustained modulation of network properties.

Previous studies described asynchronous release at many synapses in various central nervous system areas, hypothesizing its potential role in controlling the gain or excitability of postsynaptic cells by generating a smooth excitatory or inhibitory tone ([27]–[29],[31],[75]–[78] and others).

In this study, we found that the delayed and asynchronous increase of GABAergic transmission changed the computational properties of two target neuron types connected to FS interneurons, FS cells themselves and P neurons, likely promoting network desynchronization. We used in-vivo-like identical frozen noise stimuli, which is a very useful paradigm to understand the reliability and temporal precision of signal propagation between neurons. Frozen noise allows the study of spike responses independently of any stimulus-independent property of a neuron [3],[79]. In principle, the output firing of a single neuron in response to repeated frozen noise stimuli is equivalent to spike trains generated by several neurons sharing similar intrinsic properties and receiving an identical or similar sequence of complex synaptic inputs: a sort of noise-induced neuronal synchronization [79],[80]. We found that autaptic asynchronous release of GABA had a small but significant effect on the reliability of firing in FS cells, which responded to repeated noisy stimulations consistently and with sub-millisecond precision in control conditions. Importantly, most of the deterioration of spike reliability is caused by the high-frequency stimulation, which, by itself, produces prominent Ca^2+^- and/or Na^+^-dependent slow AHPs that in fact do shunt the neuronal membrane, reducing output firing properties. Nevertheless, asynchronous autaptic release from FS interneurons still led to a significant dampening of spike reliability, which added to the AHP-modulated inhibition, as evidenced by the partial restoration of spike reliability when delayed autaptic GABAergic responses were neutralized by GBZ. This GBZ-mediated effect was not due to blockade of tonic inhibition, which is known to modulate neuronal membrane gain in several brain areas [81]. Indeed, our experiments were performed using a high-flow local perfusion system, which almost completely eliminated ambient levels of GABA, as demonstrated by the lack of a GBZ-induced increase of FS-cell input resistance (Figure 7G). Our results suggest that differences in the number of autaptic connections, and thus in the magnitude of asynchronous autaptic release, might differentially amplify the AHP-dependent hyperpolarizing and shunting effect in any given FS interneuron.

Consistent with the very high spike-timing precision of FS cells, asynchronous release had no effect on jitter, but rather only controlled the output in an all-or-none fashion (reliability). This is probably due to the intrinsic and synaptic properties of FS cells that are tuned to act as very efficient coincidence detectors [15],[17].

Interestingly, asynchronous synaptic release from FS interneurons had an even more pronounced effect on postsynaptic P neurons than on autaptically connected FS cells (decreased spike reliability of 70% versus 12%, respectively). Each FS cell contacts several P neurons, actively contributing to their synchronous firing during network oscillations. The strong reduction of spike reliability and precision of P cells response to frozen noise stimulations produced by asynchronous GABA release from a presynaptic FS cell implies that P neurons are more sensitive to disorganized inhibitory events than are basket cells. This enhanced effect of asynchronous GABA release in P cells might be explained partly by the slower kinetics of their intrinsic and synaptic properties, and may act as an effective shunt of cortical excitation after intense sensory activation. Our experiments were performed in relatively quiescent cortical slices and in the constant presence of glutamate receptor antagonists. In vivo, P neurons are known to be constantly bombarded by excitatory and inhibitory synaptic activity yielding a high conductance state that has been proposed to be important for the correct integration properties and computation capabilities of cortical neurons [82]. In vivo recording of asynchronous release of GABA will therefore be necessary to reveal its functional role during specific cortical activities.

To process incoming information, cortical neurons embedded in a network utilize coding strategies relying either on the average rate of AP firing or on the exact (millisecond range) spike timing of several neurons firing synchronously [83]. Theoretical and experimental evidence indicates that cortical neurons can rely on a code based on spike timing [1],[3],[80],[84],[87], which is strictly dependent on the precision and reliability of synaptic integration [3],[79]. In the absence of millisecond-precise spike timing, network synchrony vanishes [84]. Asynchronous autaptic and synaptic release, induced by abrupt high-frequency firing of FS interneurons, reduces spike precision and reliability of cortical neurons and therefore might change the network coding strategy to propagate information from precise spike timing (such as during oscillations) to average spike rates (such as during cortical nonsynchronized states).

By switching between two modes of GABA release at their autaptic and synaptic nerve terminals, FS interneurons can effectively filter network activity, behaving as a precise coordinator when activity is normal, but quickly breaking the synchrony pattern when activity levels overshoot a certain threshold during physiological states in order to prevent/decrease generalized synchronous activity observed during epileptic discharges.

## Materials and Methods

### In Vitro Slice Preparation and Electrophysiology

All procedures were performed in accordance with Italian (Ministero della Salute), European, and United States guidelines. For electrophysiological recordings, Sprague Dawley rats or C57BL/6J mice (WT or PV^−/−^ [86]), 18–25 d old, were deeply anesthetized with isofluorane inhalation and decapitated. Brains were quickly removed and immersed in “cutting” solution (4°C) containing the following: 234 mM sucrose, 11 mM glucose, 26 mM NaHCO_3_, 2.5 mM KCl, 1.25 mM NaH_2_PO_4_, 10 mM MgSO_4_, and mM 0.5 CaCl_2_ (equilibrated with 95% O_2_–5% CO_2_). Coronal slices (300 µm) were cut with a vibratome (Leica, Nussloch, Germany) from a block of brain containing sensorimotor cortex. Slices were then incubated in oxygenated artificial cerebrospinal fluid containing the following: 126 mM NaCl, 26 mM NaHCO_3_, 2.5 mM KCl, 1.25 mM NaH_2_PO_4_, 2 mM MgSO_4_, 2 mM CaCl_2_ and 10 mM glucose (pH 7.4), initially at 32°C for 30 min, and subsequently at room temperature, before being transferred to the recording chamber. Recordings were obtained at a temperature of 30–32°C from layer V interneurons visually identified using infrared video microscopy. Basket cell interneurons were identified in current-clamp by their fast firing behavior and the absence of a large emerging apical dendrite. In all experiments, GABA_A_-receptor-mediated currents were isolated by adding 6,7-dinitroquinoxaline-2,3-dione (DNQX, 10 µM) to the bath perfusion. For most whole-cell recordings, patch-clamp electrodes (tip resistance, 2–3 MΩ) were filled with a “high Cl^–^” intracellular solution containing the following: 70 mM Kgluconate, 70 mM KCl, 2 mM NaCl, 2 mM MgCl_2_, 10 mM HEPES, 1 mM EGTA, 2 mM MgATP, and 0.3 mM Na_2_GTP (pH 7.3) corrected with KOH (290 mOsm). The estimated *E*_Cl_ was approximately –16 mV based on the Nernst equation, without correction for gluconate-generated liquid junction potential. Under these recording conditions, activation of GABA_A_ receptors resulted in inward currents at a holding potential (*V*_h_) of –70 mV. For experiments testing the effects of Ca^2+^ chelators at high concentrations, patch pipettes were filled with a similar solution: 60 mM Kgluconate, 70 mM KCl, 2 mM NaCl, 10 mM HEPES, 2 mM MgATP, and 0.3 mM Na_2_GTP, but containing either 10 mM K_4_BAPTA or 10 mM EGTA. For experiments with WT and PV^−/−^ mice, we used exactly the same low Mg^2+^ solution described in Caillard et al. [54], with Kgluconate and KCl adjusted in order to have an *E*_Cl_ of approximately −16 mV. In order to minimize washout of PV from WT cells in the whole-cell configuration, recordings were carried out right after breaking in.

For experiments exploring the effect of autaptic asynchronous release on FS-cell or postsynaptic P-cell spike precision and reliability, we used intracellular solutions with adjusted [Cl^−^] in order to match the physiological *E*_Cl_ of both FS and P cells (−72 mV and −54 mV, respectively; [47]).

Autaptic responses were detected using the high-chloride intracellular solution as previously described [22],[35]. Briefly, short depolarizing voltage steps (0.2 ms, from *V*_h_ = −70 to 10 mV) were injected into the recorded cell, in order to elicit unclamped, presumed axonal APs, producing typical GABAergic synchronous responses. These were characterized by fixed latency, high peak-amplitude fluctuation and short-term depression [22],[35]. The strength of autapses was measured by averaging the five initial responses after patch rupture. Series and membrane resistance were monitored regularly and recordings were discarded when these parameters changed significantly.

Signals were amplified, using a Multiclamp 700B patch-clamp amplifier (Axon Instruments, Foster City, California, United States), sampled at 20 kHz, filtered at 10 kHz, and stored on a PC. Data were analyzed using pClamp (Axon Instruments) and Origin (Microcal Software, Northampton, Massachusetts, United States) software. Custom written software (J. R. Huguenard) was used for analyzing spontaneous GABAergic events (Figure S1). Results are presented as mean ± standard error of the mean. Unless otherwise noted, data were statistically compared using the Student's *t*-test, and differences were considered significant if *p*<0.05.

### Quantification of Spontaneous Synaptic Events

In most cases, we used locally written software (Detector [J.R.H.]) to detect and sort sIPSCs [87] for analyzing asynchronous release (Figure S1). Briefly, individual events were detected with a threshold-triggered process from a differentiated copy of the real trace and sorted as type 1, 2, or 3 events (see Figure S1). For each cell, the detection criteria (threshold and duration of trigger for detection) were adjusted to ignore slow membrane fluctuations and electric noise while allowing maximal discrimination of sIPSCs. Detection frames were always inspected visually to ensure that the detector was working properly. To quantify activity-dependent asynchronous responses, a train stimulation protocol was run several times for each cell, and sIPSC frequency increases were normalized by calculating the ratio of event frequencies recorded in the test period (0.5–1 s) immediately after the AP trains to events recorded during the baseline period (7 s before trains). Note that in the PV^−/−^ experiments, train stimulations (200 Hz, 500 ms) were repeated 20 times in each cell, and we compared a 3-s baseline period (averaged across the 20 trials) with the time course of asynchronous response after the train (in bins of 200 ms). In general, direct detection of synaptic events was preferred to exclude any additional slower post-train effect such as AHP and, possibly, GABA_B_ autaptic responses or axonal GABA_A_-autoreceptor-mediated currents [88]. This method was also more appropriate to measure and quantify fast GABA-mediated IPSCs than the commonly used measure of charge transfer induced by spike trains [28]. However, in a subset of current-clamp experiments (Figure 2) we used the mean standard deviation (SD) of post-train noise to quantify asynchronous release of GABA. Current-clamp traces were first high-pass filtered at 10 Hz, and values were compared for 1 s before trains (baseline) versus 500 ms post-train.

In time-course experiments exploring the effect of Ca^2+^ buffers on asynchronous response (Figure 5), asynchronous sIPSC frequency increase was measured between times 0 and 60 min (50-s interval) and was normalized to the first five initial values (from time 0 to ∼4.2 min).

### Measurement of Spike Timing and Reliability in Response to Noisy In-Vivo-Like Stimuli

Input current stimuli were generated using a custom-written Octave script, by convolving traces of 3-s-long Gaussian noise with a synaptic function [3]. Signals were pre-scaled to generate current waveforms that could be injected into the recorded neurons. The root-mean-square of noise was adjusted (peak-to-peak amplitude typically 75–100 pA for FS and 75–150 pA for P cells) to yield an output firing at intermediate frequency values that were constant (±3%) across several repetitions for any given cell. This relatively low firing rate was adopted to limit the “accumulating jitter of earlier spikes” effect [3]. Under control conditions, a single noise waveform was repeatedly injected to depolarize the cell from a holding potential of −70 mV. In the case of FS cells, a high-frequency train (200–300 Hz, 500 ms) was added on top of the first half of the noise waveform in the train condition. In the case of FS–P pairs the fluctuating synaptic noise was injected in the P cell, in the presence or absence of a high-frequency train in the presynaptic FS cell (*V*_h_ = −70 mV). For all experiments, measurements of spike reliability and SD of the mean spike timing (spike jitter) were performed with Spike2 software (v5.12; Cambridge Electronic Design, Cambridge, United Kingdom). Spikes were first threshold detected, and then raster plots were constructed, and since most spikes were reliable in control conditions, we delimited areas to analyze around each peak of the peri-spike time histogram, manually adjusting the time limits in order to compare only spikes that were reliably and precisely induced in the control condition. “Spike clusters” were defined as time periods in which spikes occurred repeatedly with a reliability of at least 20% in the control condition. For example, a period in which a spike occurred only once in 30 trials (reliability = 3.3%) was removed from the analysis. We then compared the reliability and precision of APs across each noise condition to those AP clusters occurring within the same periods under control conditions. Reliability was measured as the total number of spikes occurring within single AP clusters divided by the number of trials, and for jitter analysis, spike clusters were required to be at least 20% reliable in all tested conditions (because jitter cannot be accurately measured with low numbers of spikes). Spike precision and reliability were determined for each spike cluster and then averaged across all spike clusters within selected time bins (500 ms post-train for FS-cell experiments and post-train time windows of 0–200, 200–300, and 300–500 ms for FS–P paired experiments).

## Supporting Information

Figure S1 **Automatized method for detection and sorting of sIPSCs.** (A) Voltage-clamp recording from a FS interneuron (top trace) in the continuous presence of the glutamate receptor antagonist DNQX to isolate spontaneous inhibitory GABAergic synaptic currents. Individual events were detected (vertical lines) with a threshold-triggered process from a differentiated copy of the recorded trace (bottom) and sorted as type 1, 2, or 3 events. The stimulation train artifacts were digitally removed. Detected synaptic events are color-coded. Type 1 (green) events are completely isolated events. Type 2 (blue) events are those arising from a flat baseline, but with an event on their decay phase. Type 3 (red) events are those arising on the decay of a previous event. (B and C) Same traces of (A) at faster time scales before (B) and after (C) inducing asynchronous autaptic release. Note that this approach allows accurate detection of quantal synaptic events occurring at very high frequency.
(3.57 MB TIF)

Figure S2 **The total number of APs determines the magnitude of asynchronous autaptic release.** Increasing the number of APs in the train caused proportional increase in asynchronous release (measured as SD of the mean post-train noise) regardless of their imposed frequency.
(0.64 MB TIF)

Figure S3 **Asynchronous release from FS interneurons is not due to firing from other FS cells embedded in the network.** (A) Simultaneous whole-cell (top, black traces) and cell-attached (bottom, red traces) recordings (in the continuous presence of 10 µM DNQX) from two closely spaced FS interneurons (see schematic shown in the inset at left). Single APs in the FS cell held in whole-cell induced prominent autaptic responses (shown are three consecutive, overlapped sweeps). This protocol never evoked spikes in the second FS interneurons recorded in cell-attached mode. (B) Same pair of FS interneurons as in (A). No spikes were detected in the FS neuron held in cell-attached (red, bottom) when a 0.5-s-long train at 200 Hz was induced in the FS neuron recorded in whole-cell (black, top). Note the presence of asynchronous autaptic release (black arrowheads). (C and D) Same experiment as (A) and (B) following patch rupture in the FS interneuron previously held in cell-attached. The two cells were connected by GABAergic synapses as shown by the presence of unitary synaptic responses (C). Identical high-frequency trains in the presynaptic cell induced both autaptic and synaptic asynchronous release (black and red arrowheads, respectively). AP firing in FS interneurons (both single and trains of spikes) never induced firing in nearby FS cells whether they were synaptically connected (*n* = 4) or not (*n* = 12), ruling out the possibility that increased sIPSC frequency in response to high-frequency trains is due to depolarizing GABAergic responses that would induce firing in neighboring FS interneurons. These experiments were performed using GAD67-GFP mice [64],[89] to facilitate FS interneuron recognition.
(1.55 MB TIF)

## Abbreviations

AHP: after-hyperpolarization
AP: action potential
autIPSC: autaptic inhibitory postsynaptic current
DNQX: 6,7-dinitroquinoxaline-2,3-dione
FS: fast-spiking
GBZ: gabazine
IPSC: inhibitory postsynaptic current
P: pyramidal
PV: parvalbumin
SD: standard deviation
sIPSC: spontaneous inhibitory postsynaptic current
WT: wild-type
