# Supplementary figures and images for: Desynchronization of Neocortical Networks by Asynchronous Release of GABA at Autaptic and Synaptic Contacts from Fast-Spiking Interneurons
Source: PLoS Biol. 2010 Sep 28;8(9):e1000492. doi: 10.1371/journal.pbio.1000492 (PMC2946936; doi:10.1371/journal.pbio.1000492)

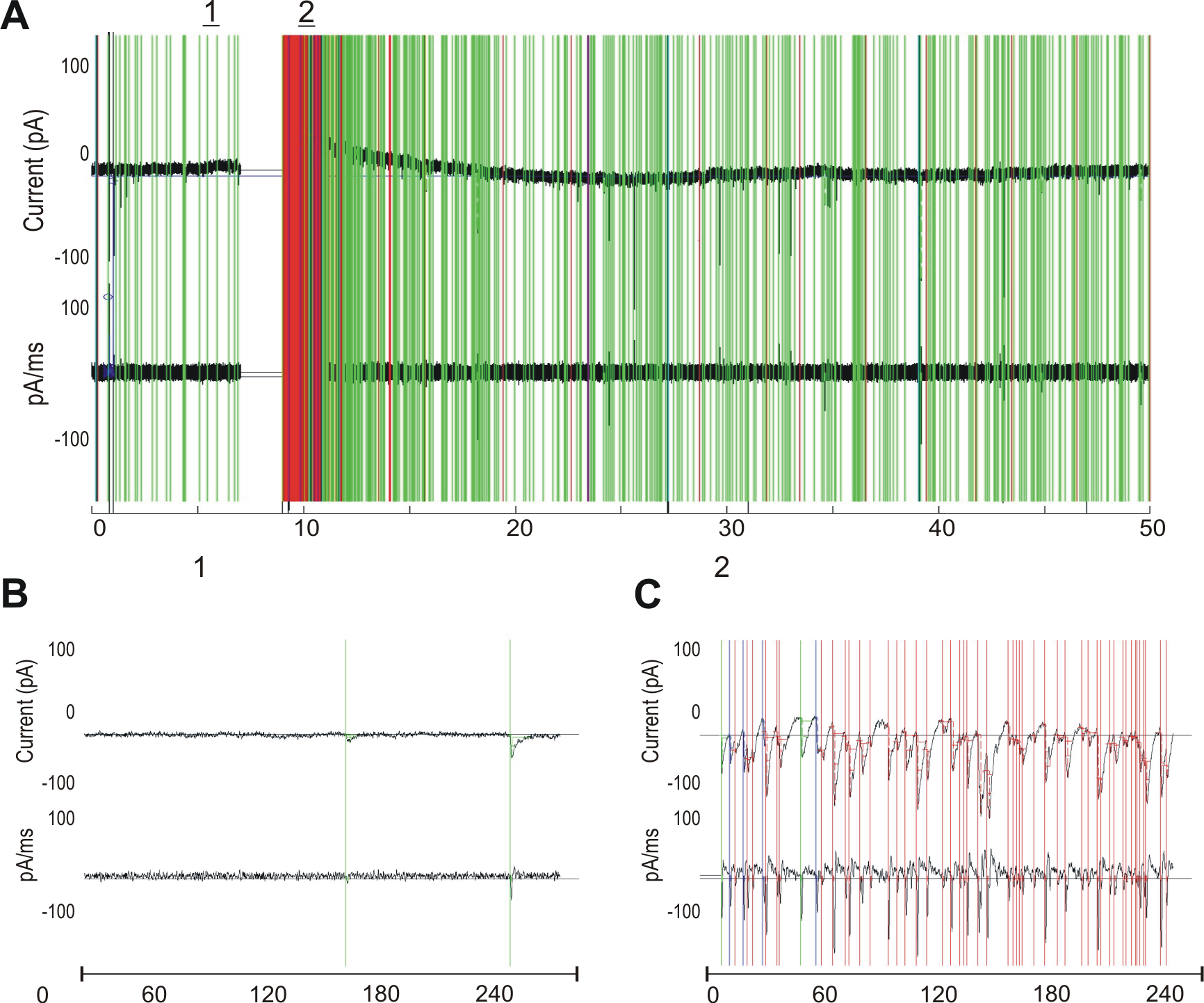

Supplement: Figure S1 — Automatized method for detection and sorting of sIPSCs. (A) Voltage-clamp recording from a FS interneuron (top trace) in the continuous presence of the glutamate receptor antagonist DNQX to isolate spontaneous inhibitory GABAergic synaptic currents. Individual events were detected (vertical lines) with a threshold-triggered process from a differentiated copy of the recorded trace (bottom) and sorted as type 1, 2, or 3 events. The stimulation train artifacts were digitally removed. Detected synaptic events are color-coded. Type 1 (green) events are completely isolated events. Type 2 (blue) events are those arising from a flat baseline, but with an event on their decay phase. Type 3 (red) events are those arising on the decay of a previous event. (B and C) Same traces of (A) at faster time scales before (B) and after (C) inducing asynchronous autaptic release. Note that this approach allows accurate detection of quantal synaptic events occurring at very high frequency. (3.57 MB TIF) [file pbio.1000492.s001.tif]

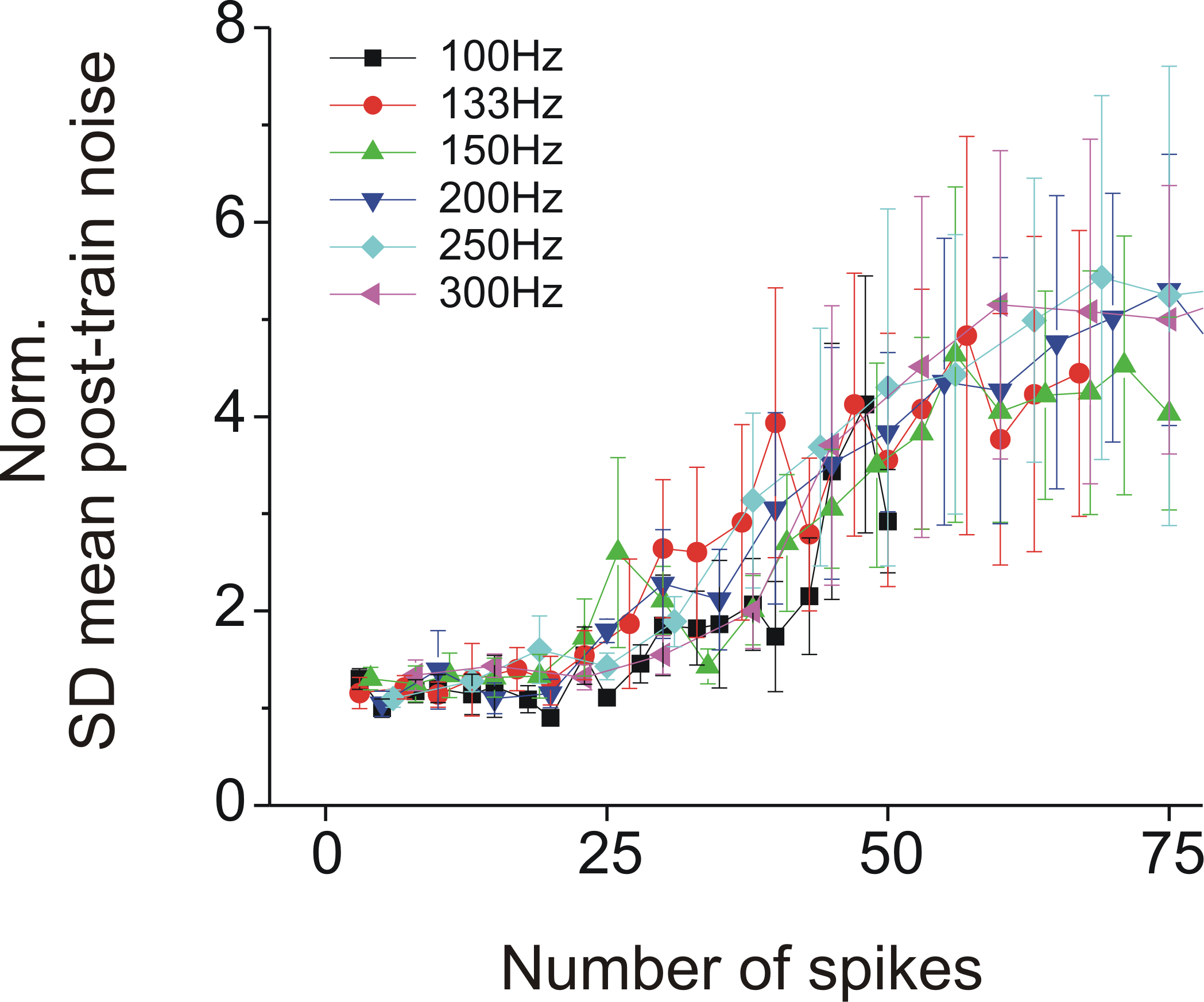

Supplement: Figure S2 — The total number of APs determines the magnitude of asynchronous autaptic release. Increasing the number of APs in the train caused proportional increase in asynchronous release (measured as SD of the mean post-train noise) regardless of their imposed frequency. (0.64 MB TIF) [file pbio.1000492.s002.tif]

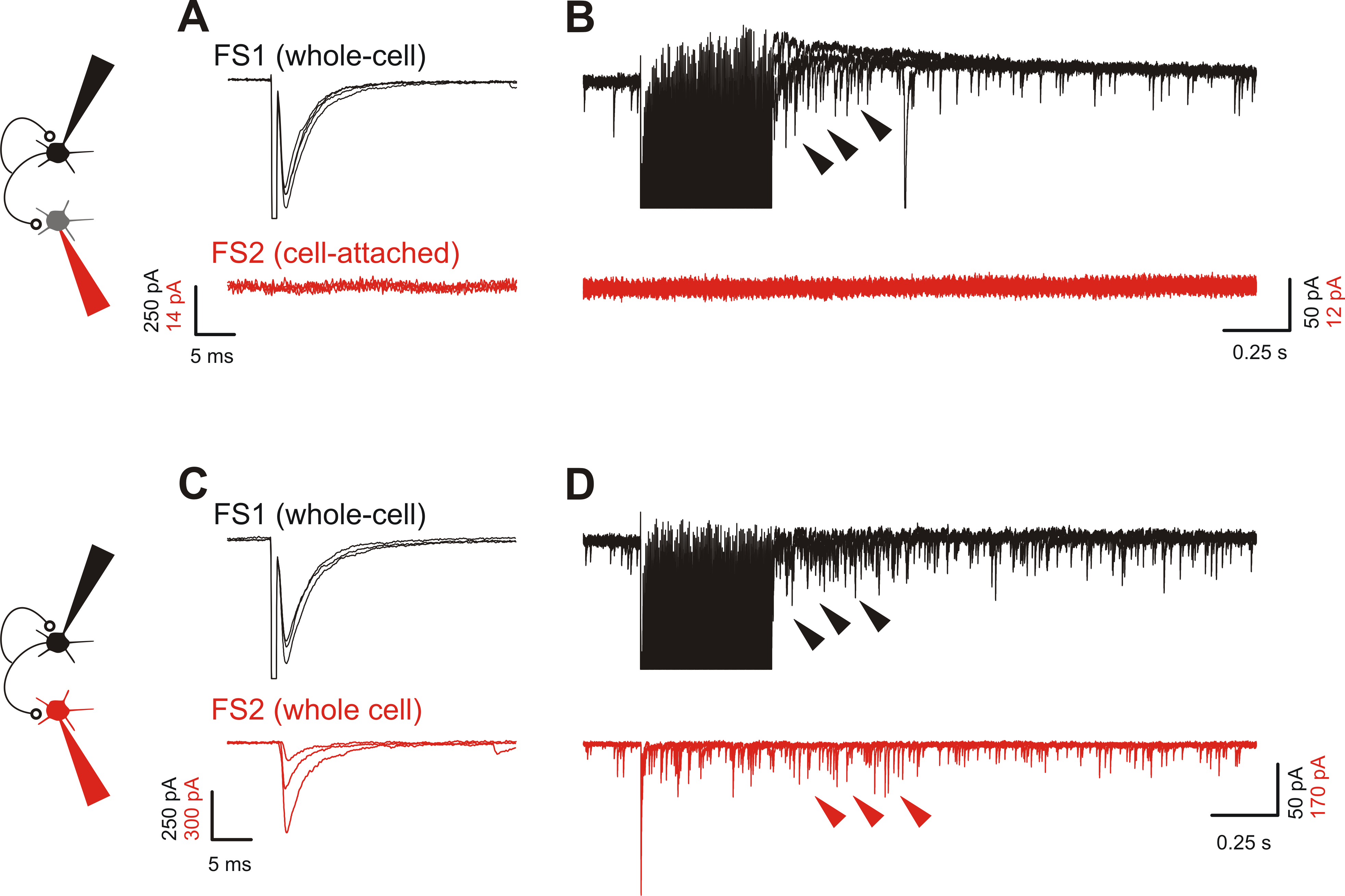

Supplement: Figure S3 — Asynchronous release from FS interneurons is not due to firing from other FS cells embedded in the network. (A) Simultaneous whole-cell (top, black traces) and cell-attached (bottom, red traces) recordings (in the continuous presence of 10 µM DNQX) from two closely spaced FS interneurons (see schematic shown in the inset at left). Single APs in the FS cell held in whole-cell induced prominent autaptic responses (shown are three consecutive, overlapped sweeps). This protocol never evoked spikes in the second FS interneurons recorded in cell-attached mode. (B) Same pair of FS interneurons as in (A). No spikes were detected in the FS neuron held in cell-attached (red, bottom) when a 0.5-s-long train at 200 Hz was induced in the FS neuron recorded in whole-cell (black, top). Note the presence of asynchronous autaptic release (black arrowheads). (C and D) Same experiment as (A) and (B) following patch rupture in the FS interneuron previously held in cell-attached. The two cells were connected by GABAergic synapses as shown by the presence of unitary synaptic responses (C). Identical high-frequency trains in the presynaptic cell induced both autaptic and synaptic asynchronous release (black and red arrowheads, respectively). AP firing in FS interneurons (both single and trains of spikes) never induced firing in nearby FS cells whether they were synaptically connected (n = 4) or not (n = 12), ruling out the possibility that increased sIPSC frequency in response to high-frequency trains is due to depolarizing GABAergic responses that would induce firing in neighboring FS interneurons. These experiments were performed using GAD67-GFP mice [64],[89] to facilitate FS interneuron recognition. (1.55 MB TIF) [file pbio.1000492.s003.tif]
